# Antibiotic prophylaxis in percutaneous nephrostomy placements and replacements for malignant urinary tract obstruction. Retrospective cohort study with systematic review and meta-analysis

**DOI:** 10.3389/fradi.2026.1787168

**Published:** 2026-05-19

**Authors:** Jeferson Rodrigo Zanon, Thiago Morais Santos, Henrique Durante, Welinton Yoshio Hirai, Marcelo Jenné Mimica, José Humberto Tavares Guerreiro Fregnani, Ricardo Dos Reis

**Affiliations:** 1 Nephrology and Palliative Care Departments of the Jales Cancer Hospital (a Barretos Cancer Hospital Unit—Pius XII Foundation), Jales, São Paulo, Brazil; 2 Interventional Radiology Department of the Jales Cancer Hospital (a Barretos Cancer Hospital Unit—Pius XII Foundation), Jales, São Paulo, Brazil; 3 Interventional Radiology Department of the Barretos Cancer Hospital (Pius XII Foundation), Barretos, São Paulo, Brazil; 4 Epidemiology and Biostatistics Center of the Barretos Cancer Hospital (Pius XII Foundation), Barretos, São Paulo, Brazil; 5 Microbiology, Pathological Sciences, and Pediatrics Departments of the Santa Casa de Misericórdia de São Paulo Medical School, São Paulo, São Paulo, Brazil; 6 Professor at the Postgraduate Program of the Barretos Cancer Hospital (Pius XII Foundation) and Superintendent of Education of the A.C. Camargo Cancer Center, São Paulo, São Paulo, Brazil; 7 Department of Gynaecological Oncology—Chair; Director of the Educational and Research Institute, and Pro-Rector of the Postgraduate Program of the Barretos Cancer Hospital (Pius XII Foundation), Barretos, São Paulo, Brazil

**Keywords:** acute kidney injury, antibiotic prophylaxis, neoplasms, nephrostomy, percutaneous, ureteral obstruction, urinary tract infections

## Abstract

**Introduction:**

Percutaneous nephrostomy (PN) can lead to urinary tract infections (UTIs), but these can be prevented by administering antibiotics. However, the effectiveness of this approach is debated.

**Methods:**

To evaluate the association between PN-related UTIs and antibiotic prophylaxis in cancer patients. This is a hybrid study was designed. A retrospective cohort study was conducted on cancer patients requiring PN, comparing antibiotic prophylaxis with no prophylaxis. The outcome, UTI, was measured up to seven days after the procedure. This cohort study was included in a systematic review and meta-analysis, for which random-effects models were used to synthesize the outcomes.

**Results:**

734 PN procedures were performed. The UTI rate following antibiotic prophylaxis was 0/158 for catheter placement and 3/66 (4.5%; *p* = 0.025) without prophylaxis. For catheter replacement, the UTI rate was 10/229 (4.4%) with prophylaxis and 2/67 (3.0%; *p* > 0.999) without. Nineteen studies with 4,606 patients were included. The binary meta-analysis included five studies and this cohort, with an odds ratio (OR) of 0.883 (95% CI = 0.400-1.951; *I*^2^ = 50.2%) for UTI. These studies had a moderate to critical risk of bias.

**Discussion:**

Antibiotics may only protect against UTIs during PN catheter placement, but this was not confirmed by the meta-analysis. A randomized clinical trial is needed.

**Conclusion:**

Antibiotics may only protect against UTIs during PN catheter placement, but this was not confirmed by the meta-analysis. A randomized clinical trial is needed.

**Systematic Review Registration:**

https://www.crd.york.ac.uk/PROSPERO/view/CRD42021231063, ClinicalTrials.gov NCT06801405 and PROSPERO CRD42021231063.

## 1 Introduction

Urinary tract obstruction has a poorly defined incidence in the oncology population, yet it accounts for approximately 7% of all cases of acute kidney injury in critically ill cancer patients (1, 2). It is not exclusive to genitourinary neoplasms and can lead to significant distress, reduced quality of life, and even reduced survival in those affected (2).

Although various techniques exist for clearing urinary obstruction, percutaneous nephrostomy (PN) is crucial in the context of anatomical changes induced by underlying malignancies, which can render traditional catheterization through the lower urinary tract impossible (3–5). Even though PN is considered to be of low technical complexity with a high success rate, it is not without complications, such as dislodgment of PN tube (24%), hematuria (15%), pain (12%), and urinary tract infection (UTI) (2, 6). In addition, cancer patients tend to keep PN in place longer with more than once catheter replacements.

Observational studies have examined UTI rates associated with PN, which incidence range from 0% (7, 8) to 70% (9) for PN placement, and from 3.0% (10) to 56.5% (11) for catheter replacements. Some of these studies date back to the late 1980s (7–9) and early 1990s (12). The methodologies vary, ranging from case series (9) to prospective cohorts (7, 8, 13). Furthermore, while one study defines urinary sepsis as fever and chills (12), others use criteria such as urine culture results and the patient's clinical condition to define a PN catheter-related UTI (10, 13–15). Additionally, the follow-up time for infection development varies, ranging from 24 h (7, 8) to 100 days (14). These results have been inconsistent due to the heterogeneity of these studies and the different definitions of PN-related UTIs, if any.

Despite this, few studies have focused on preventive measures, such as antibiotic prophylaxis (10, 12, 15). One study suggests that antibiotic prophylaxis may reduce UTI risk, but it included both oncological and non-oncological patients, which significant proportion of nephrolithiasis, and a total of 55 participants (12). Two studies have studied an oncological population only, and both concluded that antibiotic prophylaxis was not effective in preventing UTI (10, 15). There are no randomized clinical trials on this subject, even in cancer patients.

To improve our understanding of the relationship between antibiotic prophylaxis and UTI after PN (placement, and replacements), this study was designed as a hybrid of a retrospective cohort study and a systematic review with meta-analysis.

## 2 Article type

This is a hybrid study: a retrospective cohort study with systematic review and meta-analysis.

## 3 Methods

### 3.1 Part I—retrospective cohort study

After institutional review board approval, with patient informed consent waived due to participants minimal risk, medical records of patients treated for cancer who required PN were retrospectively reviewed in two hospitals of the same Foundation. A branch hospital from January 2013 to July 2020, and a main hospital from January 2014 to July 2020. This cohort study is registered on ClinicalTrials.gov website, and it was reported according to STROBE (16) guidelines.

Patients undergoing PN placement by an interventional radiologist for urinary tract clearance and subsequent catheter replacement were included. Patients and/or procedures were excluded if PN was performed to other procedures, such as anterograde placement of a double-J catheter; to treat clinical conditions other than malignant ureteral obstruction (e.g., stenosis secondary to Bricker's ileal pouch); or to clear the urinary tract of non-malignant causes (e.g., stones or non-malignant stenosis).

PN was performed under ultrasound or CT guidance, depending on the patient's clinical condition. Pigtail catheters of 8, 10, or 12 French were used for both the first placement and subsequent catheter replacements, at the discretion of the interventional radiologist. Catheter position was confirmed in all cases by pyelography with iodinated contrast injected through the catheter into the renal pelvis, guided by a surgical C-arm. The first placement was performed on an inpatient basis for patients with severe acute post-renal kidney injury, and on an outpatient basis for those with unilateral malignant urinary tract obstruction without evidence of severe UTI. Catheter replacements were routinely scheduled as outpatient procedures. When hospital admission was required—for example, for treatment of a catheter-related UTI—the replacement was performed during the inpatient stay. Except in cases of catheter obstruction, replacements were performed over a guidewire. When guidewire-assisted replacement was not feasible, a new image-guided puncture was required.

#### 3.1.1 Definitions and conduct of the study

As there is no standard definition of PN-related UTIs in the literature, definitions were established for the purpose of conducting this study and evaluating the proposed topic:

UTI occurs when there are signs and symptoms consistent with a local infection (fever, chills, dysuria, lower back pain, and costovertebral angle tenderness), such as cystitis or pyelonephritis, combined with a positive urine culture (more than 10^4^ colony-forming units of microorganisms per milliliter of urine with a catheter present or more than 10^5^ without a catheter present) (14, 17).

PN procedure-related UTI: occurs until seven days after the PN, both for catheter placement and replacement.

PN catheter-related UTI: occurs at another time, usually within 90 days of the procedure, in the presence of the PN catheter, and in the absence of another focus that would justify the infection.

Antibiotic prophylaxis was administered with a single dose of antibiotics up to 60 min before PN. Antibiotic prophylaxis was considered not given when there was no record of antibiotics in the cost center of the institution's database. For the PN placement, antibiotic prophylaxis was empirical. For catheter replacement, it was both empirical and targeted, based on pre-procedure urine culture results to guide the antibiotic choice. Procedures were excluded from analysis if patients were treated for UTI up to 7 days before or during the procedure. They were included in analysis outside the period of antibiotic prophylaxis for future UTIs.

Sociodemographic and clinical variables such as gender, race, age, underlying neoplastic disease, urine culture (see Supplementary Material), antibiotic prophylaxis and UTI were collected. Radiological data and degree of urinary obstruction were also collected. Data were stored on REDCap (18) database. For this study, PN procedures were considered independent. The sample size was assembled consecutively, non-probabilistically, and for convenience.

#### 3.1.2 Statistical analysis

Shapiro–Wilk Normality test confirmed the continuous variables non-normal distribution. These non-parametric variables are described as median and respective interquartile range (from the 25th to the 75th percentile). Mann–Whitney test was used to compare two independent samples. Categorical variables were presented in absolute and relative frequencies using chi-squared tests of association or Fisher's exact tests when the expected result was less than five in more than 20% of the cells in the association tables. *post-hoc* analysis was performed using the Bonferroni adjusted *p*-value for comparisons between more than two independent groups of categorical variables. Due to the observational nature of this study and the subsequent non-randomization of the study groups, multiple logistic regression modelling was performed to evaluate potential confounding factors. One model was created to analyze the PN catheter placement. This model was adjusted for the following variables: age, sex, neoplasm, previous UTI (occurring more than two weeks before PN), number of metastases, serum creatinine, and hospital. A second model was performed for catheter replacements, adjusted for the following variables: age, sex, neoplasm, catheter-related UTI, number of metastases, performance status, use of double-J stent, treatment received, serum creatinine and hospital. A sensitivity analysis was performed, excluding cases where no pre-procedure urine culture was collected, to establish whether missing data had influenced the results. All analyses were performed using IBM-SPSS v.26.0 software with a significant level of 5%.

### 3.2 Part II—systematic review and meta-analysis

This systematic review was reported according the PRISMA (19) guidelines, and was registered in PROSPERO. PICOS anagram was used to formulate the guiding question for cancer patients requiring PN, with or without antibiotic prophylaxis, development of UTI on observational studies (due to lack of randomized clinical trials).

#### 3.2.1 Search strategy, organization, study selection criteria, and data extraction

PubMed/MEDLINE, Cochrane Library, EMBASE, Scopus, and Web of Science databases were used for the systematic literature search. Open access theses and dissertations database was also searched. It was performed using the Boolean operators “AND” and “OR” with the index terms, and their synonyms, combining “nephrostomy, percutaneous”, “antibiotic prophylaxis”, “urinary tract infection”, “ureteral obstruction”, and “neoplasms” (details in Supplementary Material). The references of the identified studies were also assessed to identify further relevant articles (backward snowballing).

The search was performed in March 2021, with subsequent updates in June 2022, August 2023 and October 2024 during the cohort data collection period. One last update was made in March 2026. Articles found were organized using Rayyan (20) online tool, and EndNote X9 (21) software, which provided a double check to identify duplicate articles, document them, and assist with their eligibility.

Two researchers (J.R.Z. and R.D.R.) independently reviewed and filtered the articles first by titles', then by abstracts', and finally by full text's relevance. Consensus resolved disagreements. All articles found, regardless of language and publication date, were included, as were showing an association between UTI and antibiotic prophylaxis after PN in cancer patients, even if they included data on benign ureteral obstructions with a combined population (oncological and non-oncological). We excluded publications of case reports, letters, commentaries, conference abstracts, editorials, reviews, or any text without original data; animal studies; and other studies with duplicate populations (selected those involving more institutions and/or patients).

The same two authors independently extracted the following variables from the studies into a pre-defined Excel spreadsheet: study population; authors; year of publication; study country; study design; number of patients; type of neoplasm leading to urinary tract obstruction; number of PN performed; whether UTI was defined; whether antibiotic prophylaxis was given; whether the PN placement was examined, catheter replacement or both; UTI occurrence; and patient follow-up. The corresponding authors were contacted by e-mail if data were not available in the selected articles. If the requested information could not be obtained, either because the authors did not respond or because the data were not available, the article was excluded.

#### 3.2.2 Assessment of risk of bias, statistical analysis for synthesis, and outcome level of certainty

ROBINS-I (22) tool was used to assess the risk of bias, which summarizing figures were generated using robvis (23) online tool.

The inverse variance method with logit transformation in a random effects model was used to calculate the UTI pooled proportion in the single-arm meta-analysis with 95% confidence intervals. The Mantel-Haenszel method in a random-effects model was used to calculate pooled odds ratios in the binary meta-analysis with 95% confidence intervals, representing the odds of UTI with or without antibiotic prophylaxis. An odds ratio of <1 favored antibiotic prophylaxis, with *p* < 0.05 indicating statistical significance when the 95% confidence interval did not include the value one. Heterogeneity between studies was assessed using Cochran's chi-squared *Q*-test and the *I*^2^ statistic. A Chi-squared test *p* < 0.05 and *I*^2^ test >50% indicate heterogeneity. Sensitivity analysis was performed using a leave-one-out meta-analysis to assess the effect of an individual study on the pooled results. Meta-regression analyses were conducted to perform a pooled analysis of follow-up time in days that might influence the rate of UTI. Visual analysis of funnel plot symmetry and Egger's regression test, where appropriate, were used to assess publication bias. R software version 4.5.3 was used for graphical analysis and meta-analysis.

Level of certainty was assessed using the GRADE system with GRADEpro GDT online tool (24).

## 4 Results

### 4.1 Part I—retrospective cohort study

**Figure 1:**
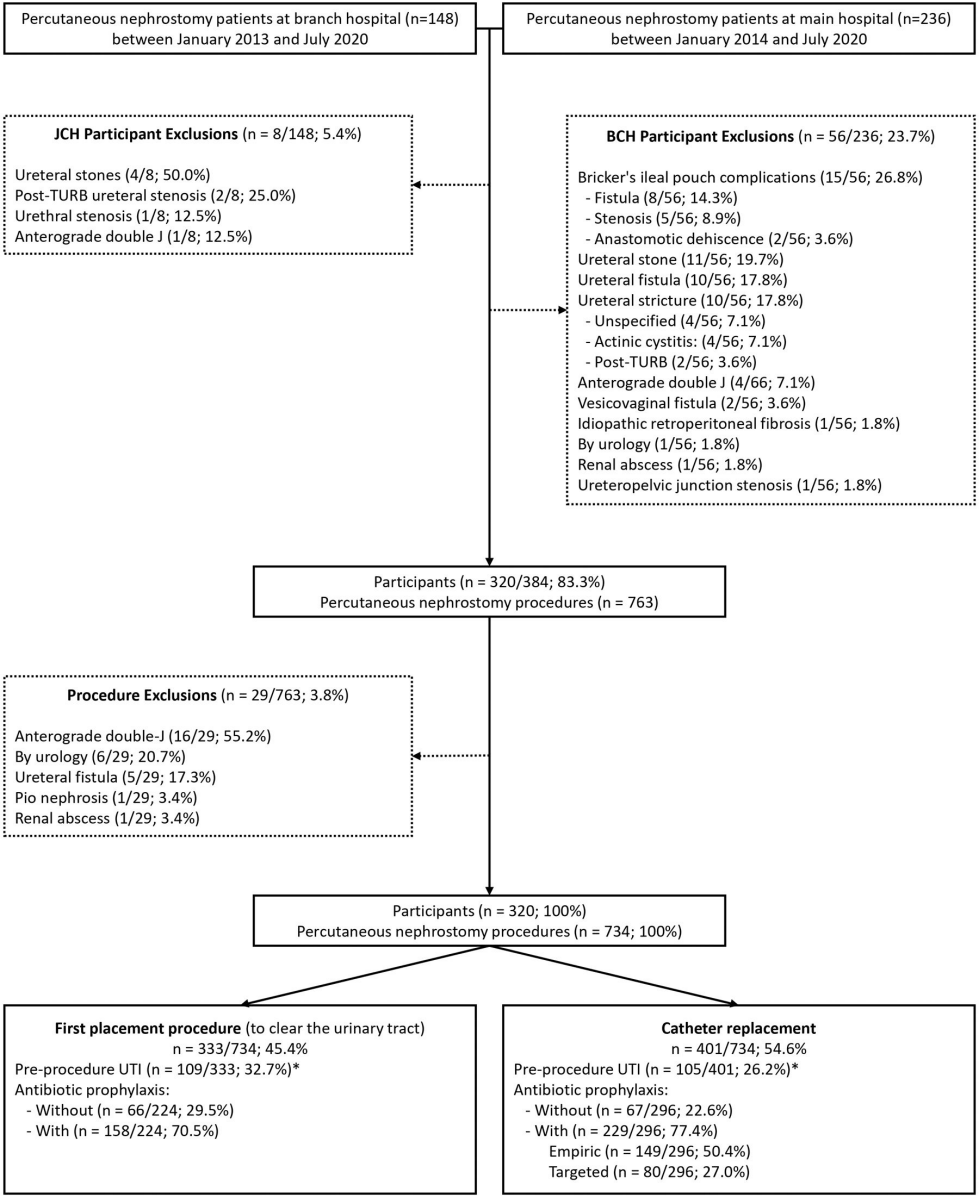
Patients undergoing percutaneous nephrostomy. TURB, transurethral resection of the bladder; JCH, Jales Cancer Hospital; BCH, Barretos Cancer Hospital; UTI, urinary tract infection; *: Procedures excluded from evaluation of procedure-related urinary tract infection after antibiotic prophylaxis because of prior antibiotic use.

Figure 1 shows the recruitment of 384 patients underwent PN over 7.5 years. These, 320 patients met the inclusion criteria for this cohort, with a total of 734 procedures performed, 333 (45.4%) for procedure first placement and 401 (54.6%) for catheter replacement.

The median (IQR) age in years of patients was 59.04 (45.51–68.79), which was significantly higher in branch hospital than in main hospital. There was no difference between these two hospitals in terms of patients' sex, skin color, neoplasms (except cervical and rectosigmoid cancer), and cancer clinical stage. Supplementary Table S1 summarizes information on participants and procedures.

To evaluate antibiotic prophylaxis for prevention of PN procedure-related UTI, 214 procedures in which patients received antimicrobial treatment for UTI prior to PN were excluded. A total of 292 urine culture samples were obtained just before or up to seven days before the 520 procedures evaluated (56.2%). Urine culture samples were obtained by natural route, indwelling catheter, or renal pelvic puncture for catheter placement. For catheter replacements, the urine culture samples were collected via a PN catheter.

The main result for urine culture samples was “negative” when collected before catheter placement (88/130; 67.7%). There was a significant difference when compared to negative urine samples collected prior to catheter replacement (10/162; 6.2%), as confirmed in the *post-hoc* analysis. *Escherichia coli* was the main microorganism found during both catheter placement (12/130; 9.2%) and catheter replacement (38/162; 23.5%), with statistical significance. The second most common microorganism found during catheter placement was *Klebsiella pneumoniae* (8/130; 6.2%) and during catheter replacement was *Pseudomonas aeruginosa* (24/162; 14.8%). A second microorganism was detected in 73 (25%) of the 292 urine culture samples collected. Supplementary Table S2 summarizes these results.

The most used antibiotic for prophylaxis was ceftriaxone for both catheter placement (116/150; 73.4%) and catheter replacement (137/229; 59.8%; *P* = 0.005 in *post-hoc* analysis). Ciprofloxacin was the second most used (21/158; 13.3% vs. 51/229; 22.3%; *P* = 0.028 in *post-hoc* analysis in both situations). The third most used antibiotic was cefazolin (18/158; 11.4%) for catheter placement, and gentamycin (15/229; 6.6%) for catheter replacement. A second antibiotic was required in 11/387 (2.8%). Supplementary Table S3 shows these results.

#### 4.1.1 PN procedure-related UTI

There was no procedure-related UTI when antibiotic prophylaxis was given at the PN catheter placement. The rate of UTI without antibiotic prophylaxis was 3/66 (4.5%), with statistical significance (*p* = 0.025). Of the three patients, one received outpatient treatment. Of the remaining two patients, both received treatment in a hospital ward. One patient died five days after completing treatment for the UTI due to underlying neoplastic disease, while the other was still alive at the end of the study.

In contrast, there was no difference in the using or not antibiotic prophylaxis to prevent procedure-related UTI for PN catheter replacement. The UTI rate after procedure with antibiotic prophylaxis was 10/229 (4.4%), and without antibiotic prophylaxis was 2/67 (3.0%), *P* > 0.999. There was also no difference in UTI rate when comparing targeted with empiric antibiotic prophylaxis as shown in Table 1. Of the two patients diagnosed with a procedure-related UTI following catheter replacement, for whom antibiotic prophylaxis had not been performed, both received treatment in a hospital ward. One patient died eight days after completing UTI treatment, while the other died seven months after completing this UTI treatment. The deaths were due to their underlying neoplastic disease.

**Table 1:** Antibiotic prophylaxis and its microorganism susceptibility, percutaneous nephrostomy and urinary tract infection.

Antibiotic prophylaxis percutaneous nephrostomy related	Urinary tract infection				P	Susceptibility of microorganisms to antibiotic prophylaxis				P
	No		Yes			No		Yes		
	N/Total	%	N/Total	%		N/Total	%	N/Total	%	
Procedure-related UTI^a^										
First placement	221/224	98.7%	3/224	1.3%	0.067^b^	16/27	59.3%	11/27	40.7%	0.023^b^
Catheter replacement	284/296	95.9%	12/296	4.1%		46/129	35.7%	83/129	64.3%	
First placement										
No antibiotic prophylaxis	63/66	95.5%	3/66	4.5%	0.025^c^	NA	NA	NA	NA	
With antibiotic prophylaxis	158/158	100%	0/158	0.0%		NA	NA	NA	NA	
Catheter replacement										
No antibiotic prophylaxis	65/67	97%	2/67	3.0%	>0.999^c^	NA	NA	NA	NA	
With antibiotic prophylaxis	219/229	95.6%	10/229	4.4%		NA	NA	NA	NA	
Empirical	142/149	95.3%	7/149	4.7%	>0.999^c^	46/49	93.9%	3/49	6.1%	<0.001^b^
Targeted	77/80	96.3%	3/80	3.7%		0/80	0.0%	80/80	100%	
Catheter-related UTI										
No antibiotic prophylaxis	255/298	85.6%	43/298	14.4%	0.478^b^	NA	NA	NA	NA	
With antibiotic prophylaxis	381/436	87.4%	55/436	12.6%		NA	NA	NA	NA	
After first placement	270/333	81.1%	63/333	18.9%	<0.001^b^	NA	NA	NA	NA	
After catheter replacement	366/401	91.3%	35/401	8.7%		NA	NA	NA	NA	

N, absolute frequency; %, relative frequency; UTI, urinary tract infection; NA, not applicable.

a Exclusion of 214 procedures due to the use of antibiotics for infection before percutaneous nephrostomy.

b Chi-squared test.

c Fisher's exact test.

The medical records show no evidence of any adverse events from prophylactic antibiotics for PN.

The results of the multiple logistic regression models did not reveal any confounding factors between the studied variables, for either the PN catheter placement or replacement. See Supplementary Material, Tables S4, S5 for more details. Sensitivity analysis revealed no differences in relation to the previously described results, as summarized in Supplementary Table S6.

Of the microorganisms detected in urine culture samples before PN placement, 16/27 (59.3%) were not susceptible to the prophylactic antibiotic used. For catheter replacements, 46/129 (35.7%) of the microorganisms detected were not susceptible to the prophylactic antibiotic used. In addition, 46/49 (93.9%) of these microorganisms were not susceptible to the prophylactic antibiotic used for empiric prophylaxis (Table 1).

#### 4.1.2 PN catheter-related UTI

Table 1 also shows that the rate of at least one episode of catheter-related UTI when antibiotic prophylaxis was not used was 43/298 (14.4%) and when antibiotic prophylaxis was used was 55/436 (12.6%), with no difference (*P* = 0.478).

### 4.2 Part II—systematic review and meta-analysis

**Figure 2:**
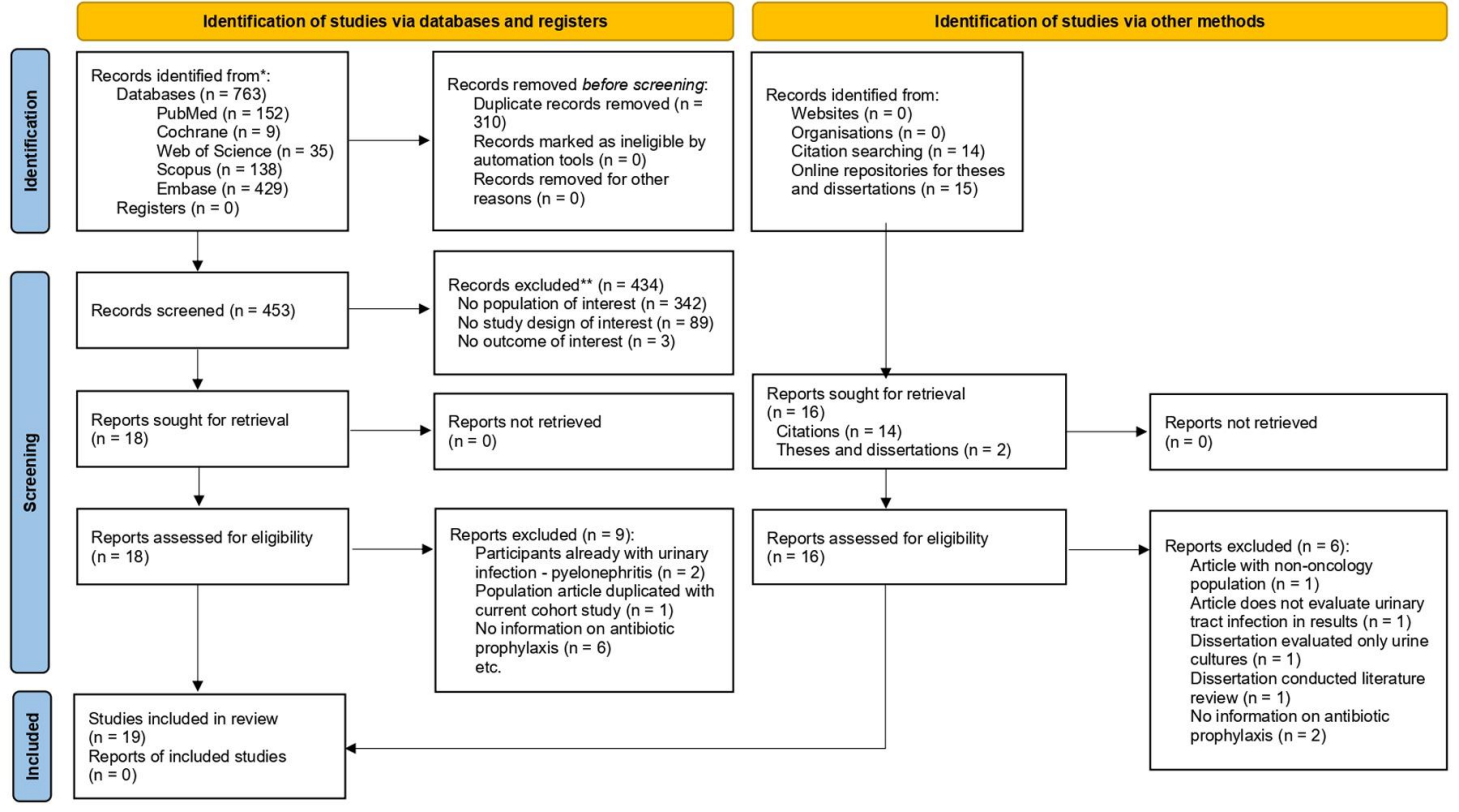
PRISMA 2020 flow diagram of the identification studies process.

The database search yielded 763 records, and the search for articles through citations and online dissertation and thesis repositories yielded an additional 29 records. Figure 2 shows the selection process, resulting in 19 articles that met the inclusion criteria for this review. Thirteen articles studied a combined population of oncological and non-oncological patients (7, 8, 12, 13, 25–33), and 6 articles the oncological population only (4, 10, 11, 14, 34, 35) (Table 2). Nine studies defined UTI, six in combined population (12, 13, 25, 28, 32, 33) and three in oncological population (4, 10, 14), although not standardized. The follow-up time in the studies evaluating UTI after PN ranged from 1 to 1,825 days, with a median of 90 days [interquartile range (IQR): 30–696]. Among studies with a combined population, the median follow-up time was 60 days (IQR: 30–547). Among studies with an exclusively oncological population, the median follow-up time was 368 days (IQR: 90–696). All the studies are observational, with most of them being retrospective cohort studies. Only three of the studies are prospective cohorts (7, 8, 13), while one is a case series study (35). Of the 19 studies, only six had percutaneous nephrostomy with antibiotic prophylaxis as their primary objective (7, 8, 10, 12, 13, 33). Two studies compared the incidence of complications between percutaneous nephrostomy and double-J catheter insertion (4, 31). The remaining 11 studies reported UTI on complications in general.

**Table 2:** Nineteen studies found on urinary tract infection and percutaneous nephrostomy.

Authors	Year	Country	Study design	Patients	Neoplasms	Nephrostomies	UTI definition	Antibiotic prophylaxis		PN placement	Catheter replacement	Follow-up time (in days)
								With	Without			
**Combined oncology and non-oncology population studies**												
Cronan JJ, *et al.* - part I	1989	US	Prospective cohort	25	NA	27	No	13	14	Yes	No	365
Cronan JJ, *et al.* - part II	1989	US	Prospective cohort	74	NA	104	No	67	37	No	Yes	1
Cochran ST, *et al.*	1991	US	Retrospective cohort	55	5	56	Yes	26	30	Yes	No	1095
Farrell TA, *et al.*	1997	US	Retrospective cohort	303	186	454	Yes	Yes	Yes	Yes	No	30
Radecka E, *et al.*	2004	Sweden	Retrospective cohort	401	153	569	No	Yes	NA	Yes	No	1825
Skolarikos A, *et al.*	2006	Greece	Retrospective cohort	530	236	650	Yes	266	384	Yes	No	30
Rana AM, *et al.*	2007	Pakistan	Retrospective cohort	667	23	765	Yes	NA	Yes	Yes	No	NA
Naeem M, *et al.*	2010	Pakistan	Retrospective cohort	200	20	20	No	Yes	NA	Yes	No	60
Matsumoto M, *et al.*	2011	Japan	Retrospective cohort	353	110	54	No	Yes	NA	Yes	No	30
Ahmad I, *et al.*	2013	Pakistan	Retrospective cohort	200	36	200	No	Yes	NA	Yes	No	90
Maramara B, *et al.*	2018	US	Retrospective cohort	71	37	71	Yes	35	36	Yes	No	60
Brick JM, *et al.*	2019	US	Retrospective cohort	126	84	493	Yes	Yes	Yes	No	Yes	730
Rizzo M, *et al.*	2021	Italy	Prospective cohort	39	70	126	Yes	NA	Yes	No	Yes	21
**Oncology population studies**												
Ku JH, *et al.*	2004	Korea	Retrospective cohort	148	NA	80	Yes	Yes	NA	No	Yes	1260
Romero FR, *et al.*	2005	Brazil	Retrospective cohort	43	5 neoplasms^a^	51	No	Yes	NA	Yes (43)	Yes (8)	696
Bahu R, *et al.*	2013	US	Retrospective cohort	200	14 neoplasms^b^	302	Yes	Yes	NA	Yes (200)	Yes (102)	90
Szvalb AD, *et al.*	2019	US	Retrospective cohort	571	More than 4 neoplasms^c^	618	Yes	Yes	NA	Yes (541)	Yes (47)	100
Alma E, *et al.*	2020	Turkey	Retrospective cohort	147	7 neoplasms^d^	229	No	Yes	NA	Yes	Yes	636
Manikandan R, *et al.*	2020	India	Case series	133	Cervix	133	No	Yes	NA	Yes	No	30

UTI, urinary tract infection; PN, percutaneous nephrostomy; US, United States; NA, not available.

a Cervix, Bladder, Prostate, Ovary, Vulva.

b Ovarian, Cervix, Prostate, Bladder, Testicle, Kidney, Colorectal, Sarcoma, Melanoma, Breast, Lung, Lymphoma, Leukaemia, Multiple Myeloma.

c Bladder and urothelial, Cervix, Prostate, Other.

d Bladder, Prostate, Colon, Cervix, Rectum, Uterus, Stomach.

All studies, including our cohort study, evaluated 4,606 patients with a total of 5,736 PN. A single-arm meta-analysis was necessary because of articles methodological and results presentations differences.

The UTI rate was 9.55% (95% CI: 4.97–17.58; *I*^2^ = 89.8%) with antibiotic prophylaxis, and 7.93% (95% CI: 1.87–28.09; *I*^2^ = 90.0%) without antibiotic prophylaxis after the PN placement. After catheter replacement, the UTI rate was 10.23% (95% CI: 1.75–42.11; *I*^2^ = 96.9%) with antibiotic prophylaxis, and 6.29% (95% CI: 3.30–11.68; *I*^2^ = 0.0%) without antibiotic prophylaxis. No significant change was observed in high heterogeneity after sensitivity analysis, subgroup analysis by population type and UTI definition, or meta-regression by patient follow-up time in the studies (Supplementary Figures S1–S4).

**Figure 3:**
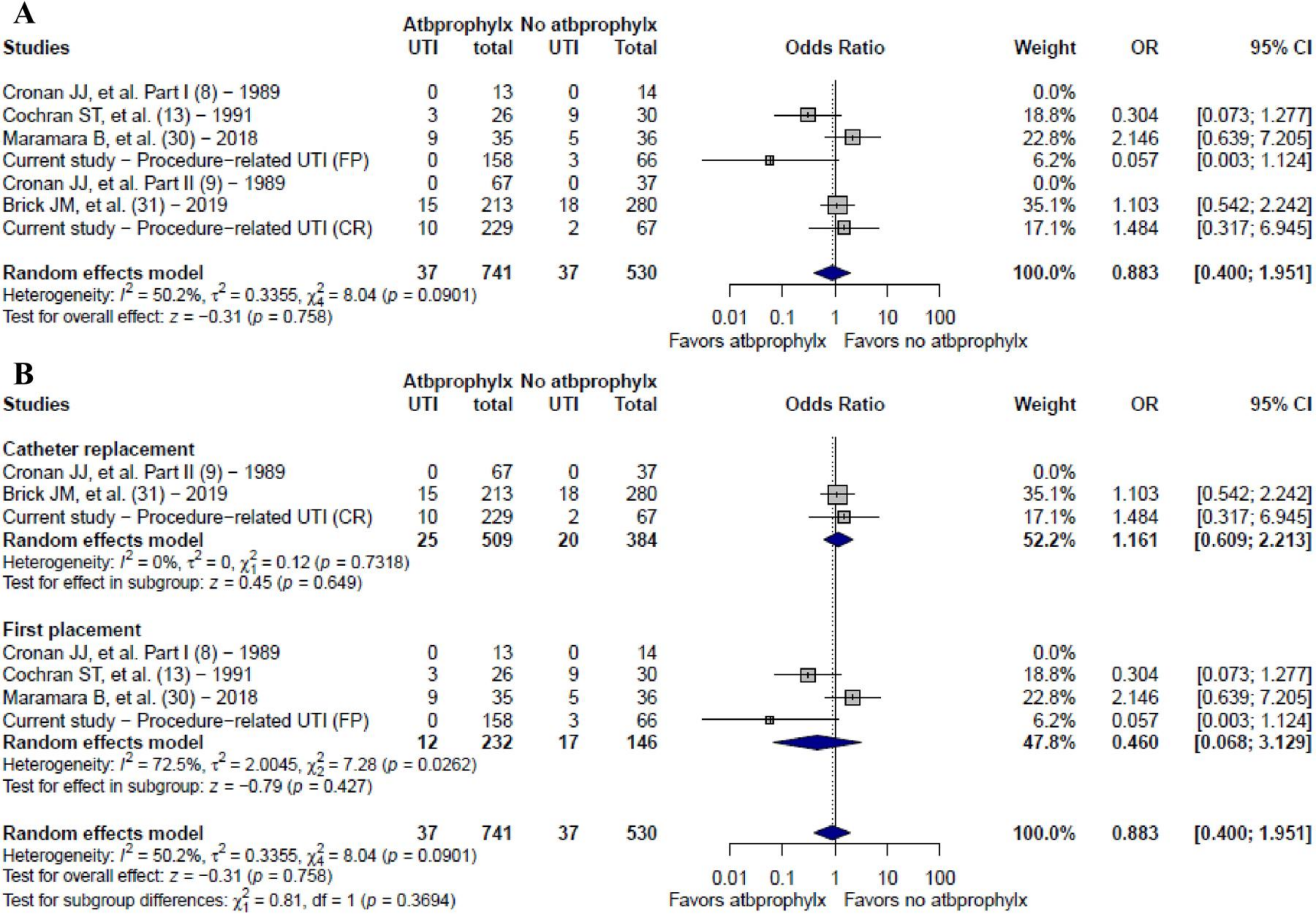
Binary meta-analysis. **(A)** Pooled OR of studies assessing UTI with and without antibiotic prophylaxis; **(B)** Pooled OR subgroup evaluation by catheter placement and replacement. Atbprophylxs: antibiotic prophylaxis; UTI, urinary tract infection; OR, odds ratio; CI, Confidence interval.

Only five studies evaluated the rate of UTI with or without antibiotic prophylaxis. These articles studied a combined population, and our cohort study evaluated this association exclusively in the oncology population. A binary meta-analysis was performed and the odds ratio found for UTI was 0.883 (95% CI: 0.400–1.951; *I*^2^ = 50.2%). The heterogeneity showed little change in the leave-one-out sensitivity test, but it was 0% in the catheter replacement subgroup (Figure 3).

Four studies had a moderate risk of bias (7, 8, 13, 33), and 15 studies had a serious (4, 10, 11, 25, 26, 28–32, 34, 35) to critical (12, 14, 27) risk of bias. Bias due to confounding in the pre-intervention assessment was the domain that dictated this result, due to the observational nature of the studies, which also explains the high heterogeneity among them (Supplementary Figure S5).

The level of certainty of the result was VERY LOW. Analyzing the subgroups of the binary meta-analysis, the level of certainty for catheter placement remains VERY LOW, but it is LOW for catheter replacement (Supplementary Table S7).

## 5 Discussion

To our knowledge, this is the largest cohort study ever conducted to evaluate antibiotic prophylaxis in the prevention of UTI following PN in cancer patients. Additionally, this study represents the first meta-analysis on this topic. Results from the cohort study suggest that antibiotic prophylaxis protects against UTI only for the PN placement, with no differences in UTI rates with or without antibiotic prophylaxis for catheter replacements. Nevertheless, our meta-analysis suggests that there is no difference in the OR for UTI after PN with or without antibiotic prophylaxis for both PN placement, and replacement.

We conducted separate analyses of UTIs at the time of PN placement and at the time of catheter replacement, because our previous study had already documented differences in urinary microbiota at these two stages of the procedure (15). This pattern persists in the current cohort. Until then, it was thought that targeted antibiotic prophylaxis for catheter replacement could protect against UTI (15). However, the current results show the opposite.

Notably, more than 90% of the microorganisms found before catheter replacement were resistant to empiric prophylaxis antibiotics. However, UTI rates did not differ between the empiric and targeted prophylaxis, or between taking or not taking antibiotic prophylaxis for catheter replacements. We can hypothesize that prior urinary colonization by the presence of the PN catheter does not influence post-procedure UTI development. Microorganisms in the catheter biofilm, not identified in urine samples, might be responsible for UTIs. A study using catheter tip cultures could help clarify these results.

Differently, almost 60% of the microorganisms found before the PN placement was resistant to the empiric antibiotic prophylaxis. Nearly 68% of the urine cultures showed no microbial growth. We can explain the statistically significant difference by addressing the binary meta-analysis results. When the population size increased by analyzing other studies, the meta-analysis showed no difference in the odds ratio for UTI between taking and not taking antibiotic prophylaxis. Furthermore, our cohort had the highest confidence interval for the odds ratio, likely due to the low UTI rates. Therefore, the issue may be methodological, or we may have a type I error (rejecting the null hypothesis when it is actually true).

It should be noted that just over 55% of urine culture samples were obtained from all the procedures evaluated, 12.1% for PN placement, and 43.6% for catheter replacement.

Our cohort showed that PN catheter-related UTI rates are higher than PN procedure-related UTI rates. Although these are not standard definitions in medical literature, studies usually assess PN catheter-related UTI, and these methodological differences between studies may explain our findings. Antibiotic prophylaxis does not protect against UTIs occurring weeks or months after the procedure, whether it's a PN placement or replacement. Our results align with Bahu and colleagues (10). Only Cronan and colleagues (8), in 1989, made it clear that the follow up to check for UTI after PN was 24 h, and therefore PN procedure-related UTI was assessed.

The result of the catheter replacement subgroup of the binary meta-analysis was the most reliable (with low heterogeneity), including three studies and our cohort, aligning with Brick and colleagues (33), and the Society of Interventional Radiology guidelines (36). According to these guidelines, antibiotic prophylaxis does not appear to be necessary before PN catheter replacement in patients with a low risk of infection according to the Cochran's criteria (12).

There is no definitive answer to the main research question of this study. We conducted a GRADE assessment to emphasize the need for further studies. The rational use of antibiotics is crucial to dealing with infections by multi-resistant organisms.

We must emphasize that, despite its limitations, this study meticulously checked and excluded from the analysis any procedures in which patients had received antibiotics prior to the procedure. We also attempted to homogenize the population by evaluating only malignant obstructions requiring PN. In this way, we believe we reduced the risk of bias between groups. We introduced two concepts, procedure- and catheter-related UTI, and demonstrated a difference in these results. Additionally, this systematic review highlights a gap in the literature on this topic and underscores the need for robust results.

Our limitations lie in the observational design of this study, which, despite all the precautions taken, still carries a high risk of selection bias in the population. Even though we didn't identify any potential confounding factors, they may still exist. We simply didn't detect them in our cohort. The sampling unit for this study was the PN procedure. As some patients underwent more than one catheter replacement procedure, certain characteristics may have been overanalyzed, particularly with regard to the age and sex of the patients in logistic regressions. We performed all the steps of the systematic review as recommended; however, we only found observational studies, with high heterogeneity between them, and with serious risk of bias, reducing the level of certainty of the results. Given the presence of rare events and studies with zero events in both arms, we did not use the Peto method, as such studies would not contribute to the pooled effect estimate. Instead, we applied a Mantel–Haenszel approach without continuity correction under a random-effects model (REML) to better account for between-study heterogeneity. However, this strategy still has limitations in the context of very sparse data, and double-zero studies contribute little information to the pooled odds ratio, which may affect the precision and robustness of our estimates.

Although our cohort showed that antibiotic prophylaxis protects against UTI after the PN placement, our meta-analysis suggests that antibiotic prophylaxis does not protect against UTI after PN at all. However, there are no data in the literature to support this approach in cancer patients due to low certainty of results. A clinical trial is needed for better understanding and patient safety. As the main bacteria found in urine cultures collected before the PN placement were *Escherichia coli*, *Pseudomonas aeruginosa* and *Enterococcus faecalis*, future studies should compare an antibiotic prophylaxis regimen involving ceftazidime and ampicillin or amikacin and ampicillin with a placebo.

## Data Availability

The datasets presented in this study can be found in online repositories. The names of the repository/repositories and accession number(s) can be found in the article/Supplementary Material.
