# Supplementary figures and images for: Antibiotic prophylaxis in percutaneous nephrostomy placements and replacements for malignant urinary tract obstruction. Retrospective cohort study with systematic review and meta-analysis
Source: Front Radiol. 2026 May 19;6:1787168. doi: 10.3389/fradi.2026.1787168 (PMC13226567; doi:10.3389/fradi.2026.1787168)

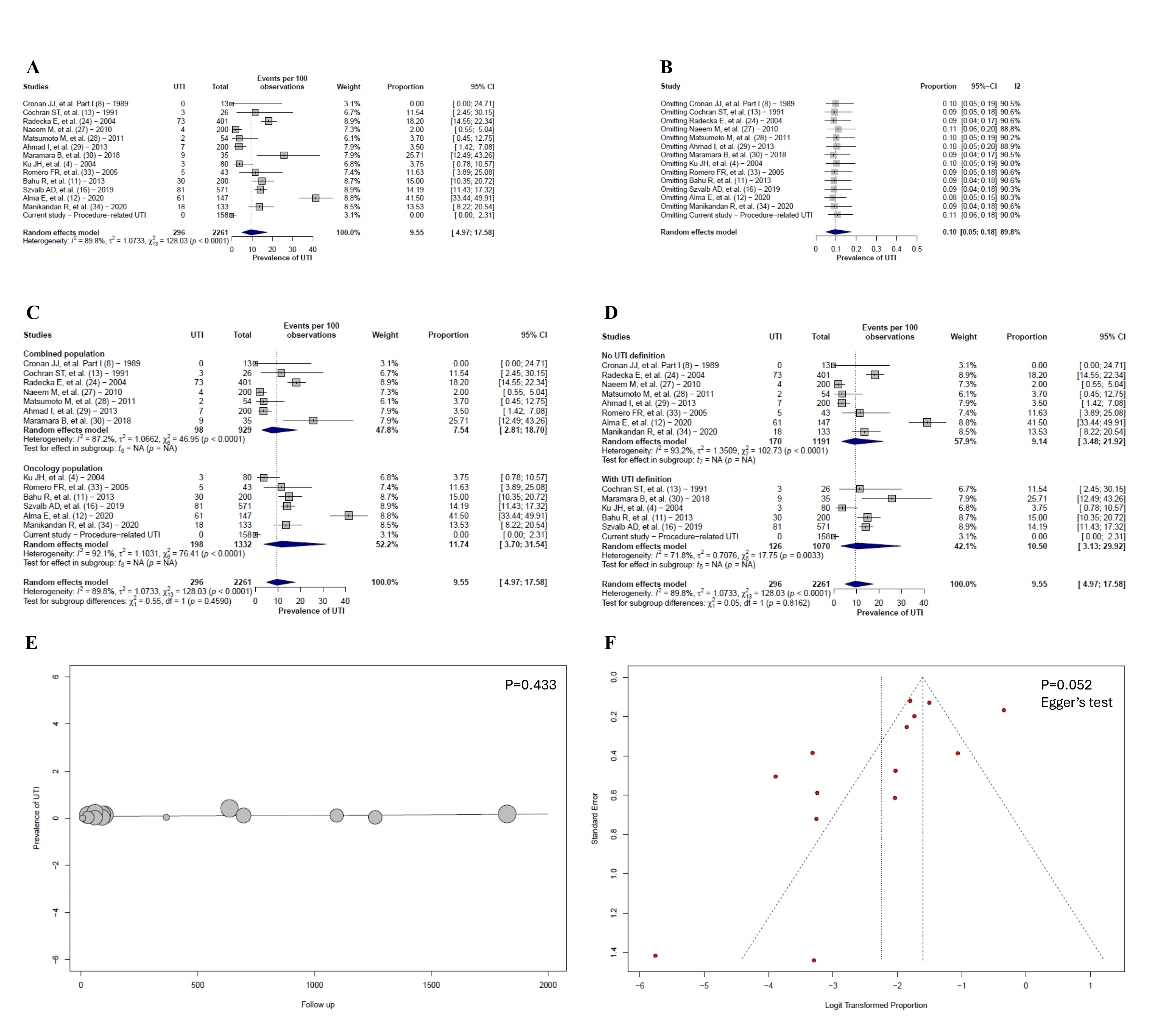

Supplement: Supplementary file 1 [file Image1.jpg]

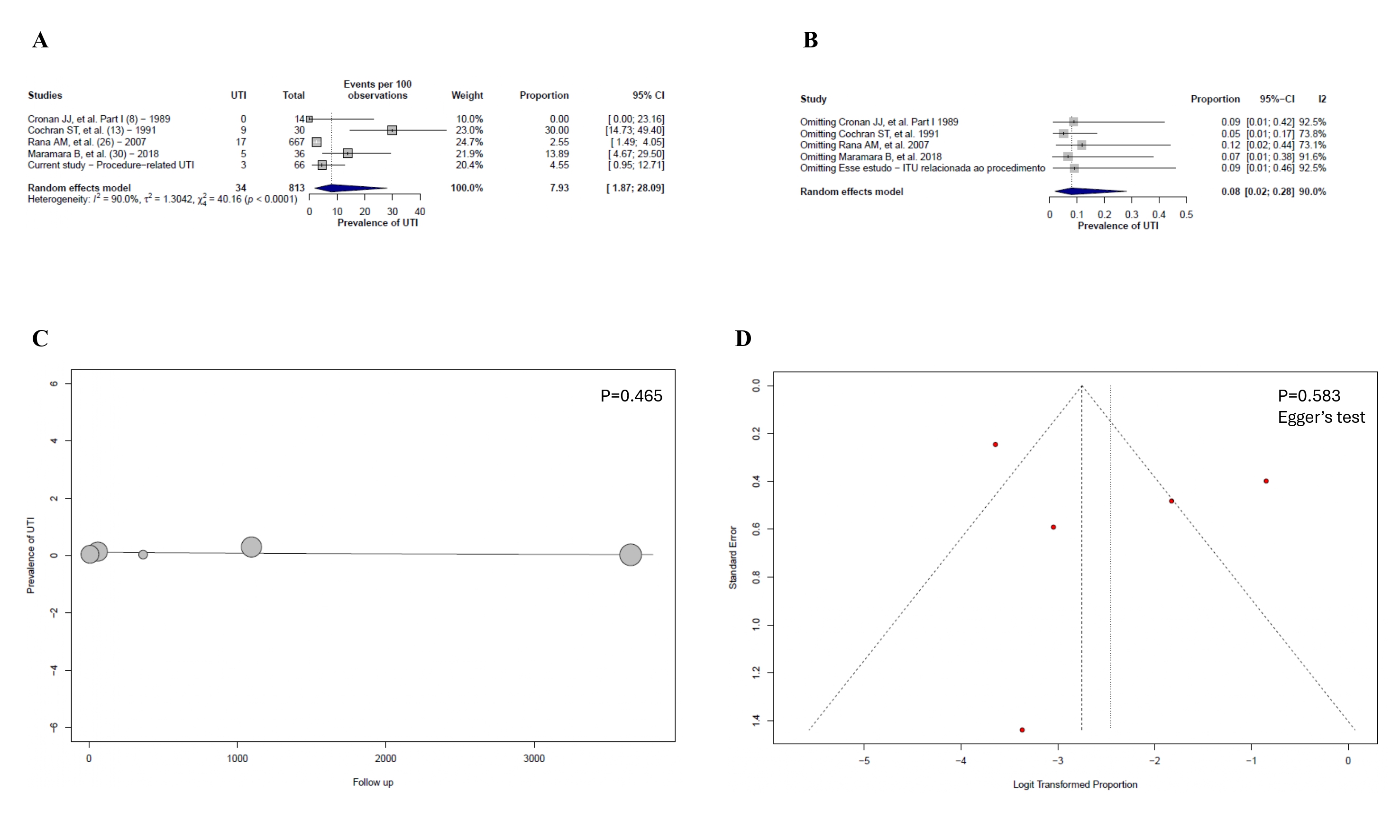

Supplement: Supplementary file 2 [file Image2.jpg]

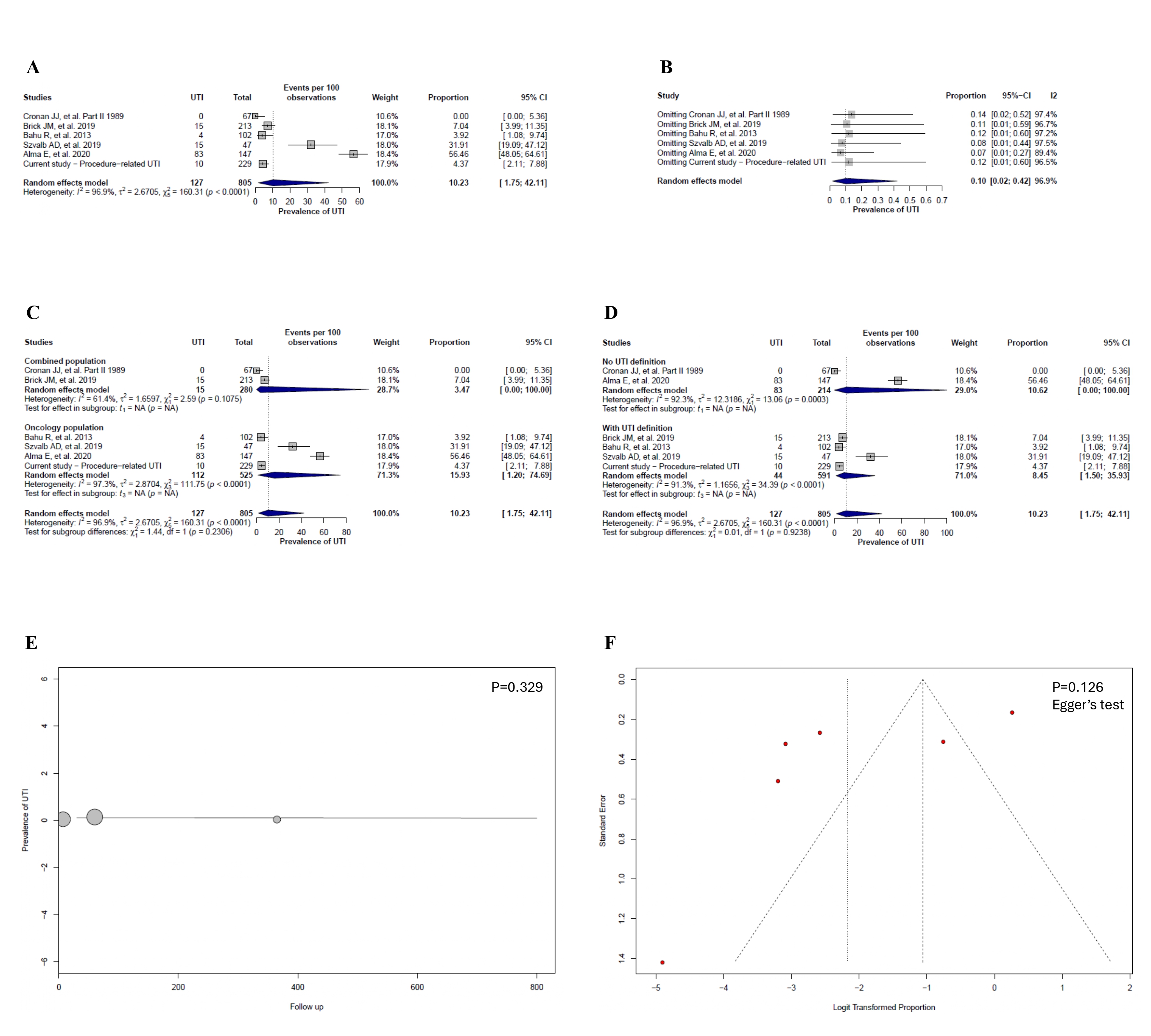

Supplement: Supplementary file 3 [file Image3.jpg]

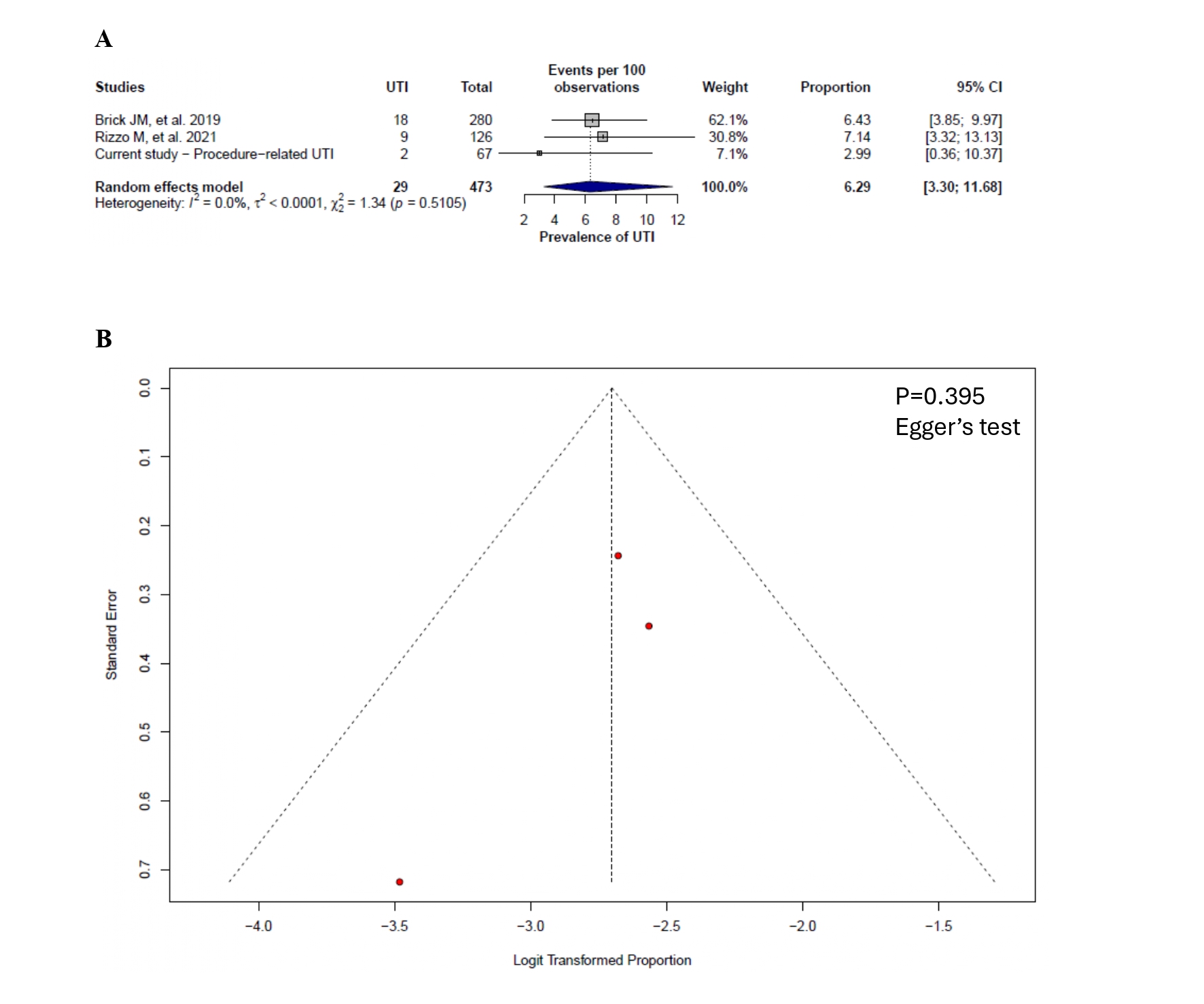

Supplement: Supplementary file 4 [file Image4.jpg]

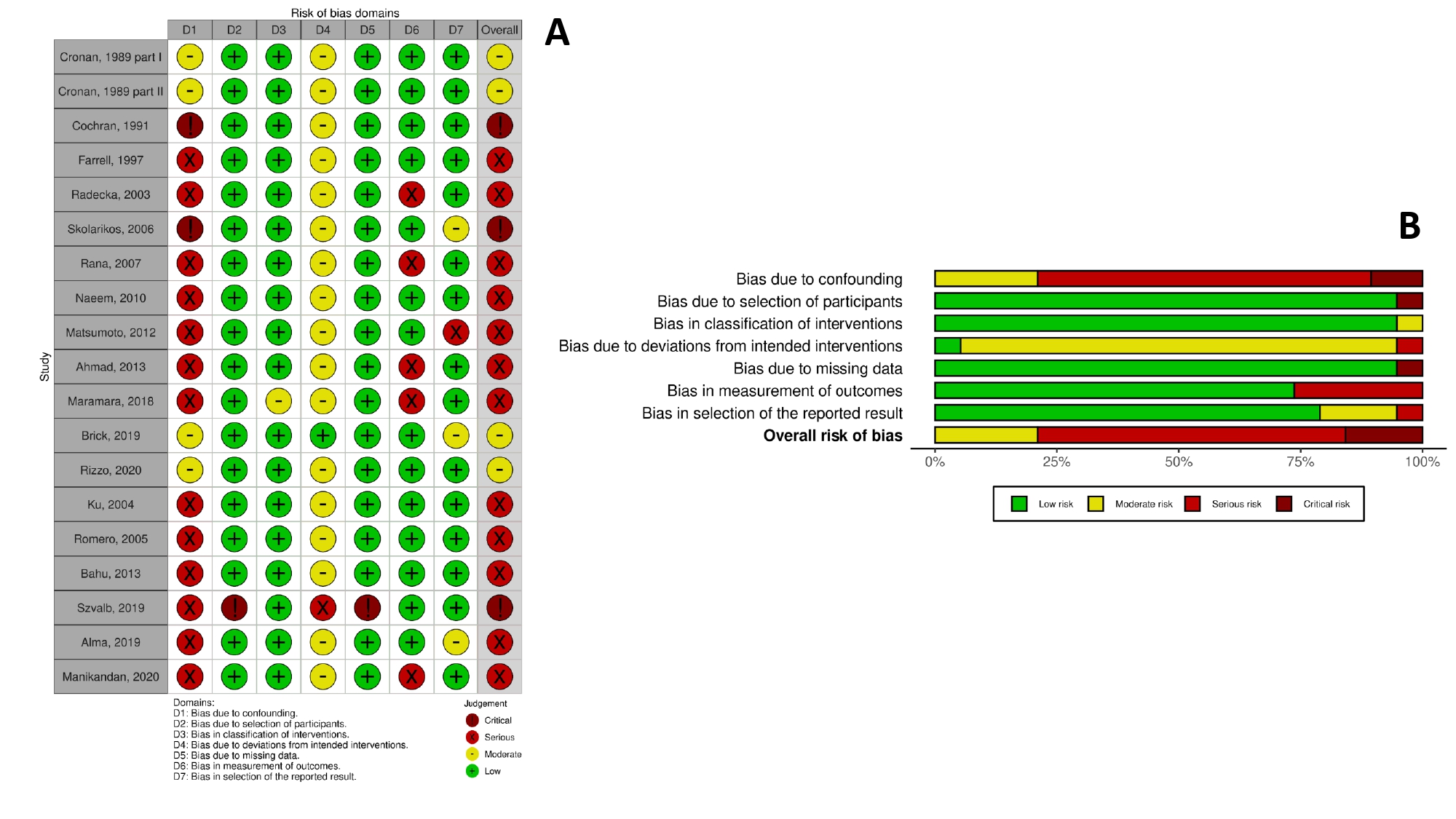

Supplement: Supplementary file 5 [file Image5.jpg]
