# Supplementary material for: Antibiotic prophylaxis in percutaneous nephrostomy placements and replacements for malignant urinary tract obstruction. Retrospective cohort study with systematic review and meta-analysis
Source: Front Radiol. 2026 May 19;6:1787168. doi: 10.3389/fradi.2026.1787168 (PMC13226567; doi:10.3389/fradi.2026.1787168)
Supplement: Supplementary file 6 [file Datasheet1.docx]

**Antibiotic prophylaxis in percutaneous nephrostomy (PN) placements and replacements for malignant urinary tract obstruction. Retrospective cohort study with systematic review and meta-analysis**

**SUPPLEMENTARY MATERIAL**

| **Table of Contents** | | |
| --- | --- | --- |
|  | | **Page** |
| Evidence before this study | | 3 |
| Assed value of this study | | 3 |
| Implications of all available evidence | | 3 |
| Online tools sites | | 4 |
| Part I – Retrospective cohort study | | 4 |
| Methodology | | 4 |
| Results | | 4 |
| **Supplementary Table 1** | Characteristics of procedures and participants who underwent percutaneous nephrostomy | 6 |
| **Supplementary Table 2** | Urine culture results prior to percutaneous nephrostomy and their relationship to empirical and targeted antibiotic prophylaxis for catheter replacements | 10 |
| **Supplementary Table 3** | Percutaneous nephrostomy antibiotic prophylaxis and its relationship to empirical and targeted antibiotic prophylaxis for catheter replacements | 13 |
| **Supplementary Table 4** | Multiple logistic regression analyses used to adjust for potential confounders in the relationship between antibiotic prophylaxis and procedure-related UTI following the PN first placement (N=224) | 14 |
| **Supplementary Table 5** | Multiple logistic regression analyses used to adjust for potential confounders in the relationship between antibiotic prophylaxis and procedure-related UTI following the PN catheter replacement (N=296) | 15 |
| **Supplementary Table 6** | Sensitivity analysis performed on antibiotic prophylaxis, percutaneous nephrostomy and urinary tract infection, excluding cases without pre-procedure urine cultures | 16 |
| Part II – Systematic review and meta-analysis | | 17 |
| Methodology | | 17 |
| Search strategy | | 17 |
| Results | | 19 |
| Reports excluded by eligibility criteria | | 19 |
| Via databases | | 19 |
| Via other methods | | 20 |
| Single-arm meta-analysis results | | 21 |
| UTI rate after percutaneous nephrostomy placement with antibiotic prophylaxis | | 21 |
| UTI rate after percutaneous nephrostomy placement without antibiotic prophylaxis | | 21 |
| UTI rate after catheter replacement with antibiotic prophylaxis | | 22 |
| UTI rate after catheter replacement without antibiotic prophylaxis | | 22 |
| Risk of bias | | 22 |
| Level of certainty of results | | 23 |
| **Supplementary Table 7** | The level of certainty of the result determined by the GRADE assessment | 24 |
| References | | 26 |
| Abbreviations on main text, tables, and figures | | 27 |
| Supplementary tables and figures | | 28 |

**Evidence before this study**

The use of antibiotics has undoubtedly been a major milestone in the history of modern medicine. However, their indiscriminate use has had serious consequences for daily medical practice. In addition to unwanted side effects, the development of multi-resistant bacteria, especially in the hospital environment, is a villain to be fought. In this context, the prevention of infections, including after potentially contaminated procedures, is of paramount importance. But does antibiotic prophylaxis really prevent urinary tract infections in less complex urological procedures?

There is a Cochrane review on indwelling urinary catheters (1) and a few randomized clinical trials on antibiotic prophylaxis for nephrolithotripsy and shock wave nephrolithotripsy (2-8), but nothing as robust for percutaneous nephrostomy. The studies that have been done are observational, methodologically heterogeneous, and have very different rates of urinary tract infection. There are no randomized clinical trials.

**Assed value of this study**

To our knowledge, this is the first systematic review of antibiotic prophylaxis for percutaneous nephrostomy, specifically in the oncology population, with a meta-analysis of these observational studies. To our knowledge, our study includes one of the largest, if not the largest, cohort of cancer patients ever studied on this topic. In addition, we evaluated the relationship between antibiotic prophylaxis and procedure- and catheter-related urinary tract infections, both after initial placement and after replacement of these nephrostomy catheters. This is unprecedented in the medical literature.

**Implications of all available evidence**

Based on the results obtained, it appears that antibiotic prophylaxis does not protect against urinary tract infection after percutaneous nephrostomy, especially after catheter replacement. Reduced use of antibiotics in clinical practice, even for prophylaxis, may contribute to reduce multi-resistant bacteria incidence. However, because of the high risk of bias in the observational studies found, including our cohort study, a randomized clinical trial is needed to better answer this research question and to promote better safety for oncology and non-oncology patients.

**Online tools sites**

- REDCap (Research Electronic Data Capture) database: <https://hcbredcap.com.br/>
- ClinicalTrials.gov: <https://www.clinicaltrials.gov>
- PROSPERO (International Prospective Register of Systematic Reviews): <https://www.crd.york.ac.uk/prospero/>
- Rayyan: <https://www.rayyan.ai/>
- Robvis: <https://mcguinlu.shinyapps.io/robvis/>
- GRADEpro: <https://gdt.gradepro.org/app/#projects>
- DeepL: <https://www.deepl.com/write>
- Mendeley Data: <https://data.mendeley.com>

**Part I – Retrospective cohort study**

**Methodology**

Urine culture samples were obtained by natural route, indwelling catheter, or renal pelvic puncture for catheter first placement. For catheter replacements, the urine culture samples were collected via a nephrostomy catheter.

For the analysis of potential confounders, comorbidities associated with neoplastic disease were not evaluated because it would not be possible to constantly monitor patients' blood glucose and blood pressure levels in a retrospective study. As most participants had metastatic neoplastic disease, progression to cachexia was inevitable. This would alter blood glucose and blood pressure levels, eliminating the need for continuous hypoglycemic and antihypertensive medication. Therefore, these two comorbidities would not influence any other clinical condition except for the cancer itself. The operator factor was not evaluated as only two professionals were available to perform the procedure in the main hospital and only one professional was available in the branch hospital during the study period.

**Results**

Supplementary Table 1 supplement summarizes all information on participants and procedures.

Supplementary Table 2 summarizes urine culture samples results related to percutaneous nephrostomy first placement, catheter replacement, and empiric and targeted antibiotic prophylaxis. As well as Supplementary Table 3 for antibiotic used to prophylaxis

Supplementary Table 4 summarizes the results of the multiple logistic regression modelling performed to analyze the factors that could potentially confound the results of the analysis of antibiotic prophylaxis at PN catheter placement. This analysis was adjusted for age, sex, type of neoplasm, previous UTI (occurring more than two weeks before PN catheter placement), number of metastases, serum creatinine, and hospital.

Similarly, Supplementary Table 5 summarizes the results of multiple logistic regression modelling performed to evaluate potential confounding factors in the analysis of antibiotic prophylaxis at catheter replacements. This was adjusted for age, sex, type of neoplasm, catheter-related UTI, number of metastases, performance status, use of a double-J stent, treatment, serum creatinine and hospital.

Supplementary Table 6 summarizes the results of a sensitivity analysis that excluded cases where no pre-procedure urine culture was collected.

| **Supplementary Table 1.** Characteristics of procedures and participants who underwent percutaneous nephrostomy | | | | | | | | | | | | |
| --- | --- | --- | --- | --- | --- | --- | --- | --- | --- | --- | --- | --- |
|  | **Both hospitals** | | |  | **Branch hospital** | | | **Main hospital** | | | **P** | **Post hoc^¶^** |
| **Patients** | Total | 𝑥 ̃/N | IQR/% |  | Total | 𝑥 ̃/N | IQR/% | Total | 𝑥 ̃/N | IQR/% |  |  |
| Age (in years old) | 320 | 59.04 | 45.51-68.79 |  | 140 | 63.95 | 52.16-73.63 | 180 | 54.63 | 39.85-64.39 | <0.001** |  |
| Survival with disease (in days) | 320 | 528.5 | 293.5-1160 |  | 140 | 563 | 303-1355.5 | 180 | 516 | 284-1111.5 | 0.306** |  |
| Time with nephrostomy (in days) | 320 | 105 | 49-219 |  | 140 | 96 | 56-196 | 180 | 113 | 45-222.5 | 0.706** |  |
| Sex |  |  |  |  |  |  |  |  |  |  | 0.775^††^ |  |
| Female | 320 | 188 | 58.8% |  | 140 | 81 | 57.9% | 180 | 107 | 59.4% |  |  |
| Male | 320 | 132 | 41.3% |  | 140 | 59 | 42.1% | 180 | 73 | 40.6% |  |  |
| Skin colour* |  |  |  |  |  |  |  |  |  |  | 0.150^††^ |  |
| Not white | 317 | 108 | 35.4% |  | 138 | 41 | 29.7% | 179 | 67 | 37.4% |  |  |
| White | 317 | 209 | 64.6% |  | 138 | 97 | 70.3% | 179 | 112 | 62.6% |  |  |
| Neoplasms |  |  |  |  |  |  |  |  |  |  | 0.059^‡‡^ |  |
| Cervix | 320 | 130 | 40.6% |  | 140 | 47 | 33.3% | 180 | 83 | 46.1% |  | 0.021 |
| Prostate | 320 | 58 | 18.1% |  | 140 | 28 | 20% | 180 | 30 | 16.7% |  | 0.423 |
| Urinary bladder | 320 | 52 | 16.3% |  | 140 | 22 | 15.7% | 180 | 30 | 16.7% |  | 0.841 |
| Rectosigmoid | 320 | 28 | 8.8% |  | 140 | 20 | 14.3% | 180 | 8 | 4.4% |  | 0.002 |
| Endometrium | 320 | 13 | 4.1% |  | 140 | 7 | 5% | 180 | 6 | 3.3% |  | 0.484 |
| Colon | 320 | 9 | 2.8% |  | 140 | 5 | 3.6% | 180 | 4 | 2.2% |  | 0.484 |
| Sarcoma | 320 | 6 | 1.9% |  | 140 | 1 | 0.7% | 180 | 5 | 2.8% |  | 0.162 |
| Ovary | 320 | 5 | 1.6% |  | 140 | 3 | 2.1% | 180 | 2 | 1.1% |  | 0.484 |
| Vagina | 320 | 3 | 0.9% |  | 140 | 1 | 0.7% | 180 | 2 | 1.1% |  | 0.690 |
| Uterus | 320 | 3 | 0.9% |  | 140 | 2 | 1.4% | 180 | 1 | 0.6% |  | 0.423 |
| Lymphoma | 320 | 3 | 0.9% |  | 140 | 0 | 0% | 180 | 3 | 1.7% |  | 0.134 |
| Testicle | 320 | 2 | 0.6% |  | 140 | 1 | 0.7% | 180 | 1 | 0.6% |  | 0.841 |
| Unknown primary | 320 | 2 | 0.6% |  | 140 | 1 | 0.7% | 180 | 1 | 0.6% |  | 0.841 |
| Urethra | 320 | 1 | 0.3% |  | 140 | 0 | 0% | 180 | 1 | 0.6% |  | 0.368 |
| Stomach | 320 | 1 | 0.3% |  | 140 | 1 | 0.7% | 180 | 0 | 0% |  | 0.271 |
| Pancreas | 320 | 1 | 0.3% |  | 140 | 1 | 0.7% | 180 | 0 | 0% |  | 0.271 |
| Multiple myeloma | 320 | 1 | 0.3% |  | 140 | 0 | 0% | 180 | 1 | 0.6% |  | 0.368 |
| Melanoma | 320 | 1 | 0.3% |  | 140 | 0 | 0% | 180 | 1 | 0.6% |  | 0.368 |
| Breast cancer | 320 | 1 | 0.3% |  | 140 | 0 | 0% | 180 | 1 | 0.6% |  | 0.368 |
| Clinical stage^†^ |  |  |  |  |  |  |  |  |  |  | 0.972^‡‡^ |  |
| I | 318 | 1 | 0.3% |  | 139 | 0 | 0% | 179 | 1 | 0.6% |  | 0.368 |
| II | 318 | 3 | 0.9% |  | 139 | 1 | 0.7% | 179 | 2 | 1.1% |  | 0.689 |
| III | 318 | 52 | 16.4% |  | 139 | 22 | 15.8% | 179 | 30 | 16.8% |  | 0.841 |
| IV | 318 | 262 | 82.4% |  | 139 | 116 | 83.5% | 179 | 146 | 81.6% |  | 0.689 |
| Diagnosis of urinary tract obstruction in the context of cancer treatment |  |  |  |  |  |  |  |  |  |  | 0.002^††^ |  |
| Before cancer treatment | 320 | 163 | 50.9% |  | 140 | 68 | 48.6% | 180 | 95 | 52.8% |  | 0.484 |
| During cancer treatment | 320 | 134 | 41.9% |  | 140 | 54 | 38.6% | 180 | 80 | 44.4% |  | 0.271 |
| Palliative care | 320 | 23 | 7.2% |  | 140 | 18 | 12.9% | 180 | 5 | 2.8% |  | <0.001 |
| Urinary obstruction diagnosis |  |  |  |  |  |  |  |  |  |  | 0.010^††^ |  |
| Ultrasound | 320 | 98 | 30.6% |  | 140 | 39 | 27.9% | 180 | 59 | 32.8% |  | 0.368 |
| Computed tomography scan | 320 | 176 | 55% |  | 140 | 89 | 63.6% | 180 | 87 | 48.3% |  | 0.006 |
| Magnetic resonance imaging | 320 | 45 | 14.1% |  | 140 | 12 | 8.6% | 180 | 33 | 18.3% |  | 0.012 |
| Intra-operative diagnosis | 320 | 1 | 0.3% |  | 140 | 0 | 0% | 180 | 1 | 0.6% |  | 0.368 |
|  |  |  |  |  |  |  |  |  |  |  |  |  |
| Patient status at end of study |  |  |  |  |  |  |  |  |  |  | 0.283^††^ |  |
| Alive without disease | 320 | 18 | 5.6% |  | 140 | 7 | 5% | 180 | 11 | 6.1% |  | 0.689 |
| Alive with disease | 320 | 29 | 9.1% |  | 140 | 15 | 10.7% | 180 | 14 | 7.8% |  | 0.368 |
| Death from cancer | 320 | 242 | 75.6% |  | 140 | 108 | 77.1% | 180 | 134 | 74.4% |  | 0.549 |
| Death from other causes | 320 | 26 | 8.1% |  | 140 | 10 | 7.1% | 180 | 16 | 8.9% |  | 0.549 |
| Loss to follow-up | 320 | 5 | 1.6% |  | 140 | 0 | 0% | 180 | 5 | 2.8% |  | 0.046 |
| **Percutaneous nephrostomies** |  |  |  |  |  |  |  |  |  |  |  |  |
| First placement | 734 | 333 | 45.4% |  | 319 | 146 | 45.8% | 415 | 187 | 45.1% | 0.849^††^ |  |
| Catheter replacement | 734 | 401 | 54.6% |  | 319 | 173 | 54.2% | 415 | 228 | 54.9% |  |  |
| Degree of hydronephrosis^‡^ |  |  |  |  |  |  |  |  |  |  | 0.017^††^ |  |
| Mild | 326 | 17 | 5.2% |  | 141 | 4 | 2.8% | 185 | 13 | 7% |  | 0.089 |
| Moderate | 326 | 178 | 54.6% |  | 141 | 69 | 48.9% | 185 | 109 | 58.9% |  | 0.072 |
| Severe | 326 | 131 | 40.2% |  | 141 | 68 | 48.3% | 185 | 63 | 34.1% |  | 0.009 |
| Catheter side |  |  |  |  |  |  |  |  |  |  | 0.144^††^ |  |
| Right | 734 | 209 | 28.5% |  | 319 | 92 | 28.8% | 415 | 117 | 28.2% |  | 0.841 |
| Left | 734 | 257 | 35% |  | 319 | 100 | 31.3% | 415 | 157 | 37.8% |  | 0.072 |
| Both | 734 | 268 | 36.5% |  | 319 | 127 | 39.8% | 415 | 141 | 34% |  | 0.110 |
| Catheter gauge^§^ |  |  |  |  |  |  |  |  |  |  | 0.712^††^ |  |
| 8 French^‖^ | 657 | 449 | 68.3% |  | 293 | 205 | 70% | 364 | 244 | 67% |  | 0.424 |
| 10 French | 657 | 193 | 29.4% |  | 293 | 82 | 28% | 364 | 111 | 30.5% |  | 0.484 |
| 12 French | 657 | 15 | 2.3% |  | 293 | 6 | 2% | 364 | 9 | 2.5% |  | 0.690 |
| Cancer treatment status at time of procedure |  |  |  |  |  |  |  |  |  |  | 0.016^††^ |  |
| Chemotherapy treatment | 734 | 225 | 30.7% |  | 319 | 106 | 33.2% | 415 | 119 | 28.7% |  | 0.194 |
| Radiotherapy | 734 | 32 | 4.4% |  | 319 | 10 | 3.1% | 415 | 22 | 5.3% |  | 0.162 |
| Chemoradiotherapy | 734 | 27 | 3.7% |  | 319 | 6 | 1.9% | 415 | 21 | 5.1% |  | 0.021 |
| Palliative care | 734 | 101 | 13.8% |  | 319 | 53 | 16.6% | 415 | 48 | 11.6% |  | 0.046 |
| Follow-up and/or hormone blockade | 734 | 349 | 47.5% |  | 319 | 144 | 45.1% | 415 | 205 | 49.4% |  | 0.271 |
| Presence of fistula to the urinary tract |  |  |  |  |  |  |  |  |  |  |  |  |
| Yes | 734 | 141 | 19.2% |  | 319 | 254 | 79.6% | 415 | 339 | 81.7% | 0.482^††^ |  |
| No fistula | 734 | 593 | 80.8% |  | 319 | 65 | 20.4% | 415 | 76 | 18.3% |  |  |
| N: absolute frequency; %: relative frequency; 𝑥 ̃: median; IQR: interquartile range. | | | | | | | | | | | | |
| *: three missing data; †: two missing data; ‡: seven missing data; §: 77 missing data; ‖: 1 French, which defines the caliber of the catheter as 0.66 millimeters; ¶: p-value adjusted by the Bonferroni method and individually calculated from the value of the standardized residuals adjusted in the cross-tabulation; **: Mann-Whitney test; ††: Chi-square test; ‡‡: Fisher's exact test | | | | | | | | | | | | |

| **Supplementary Table 2.** Urine culture results prior to percutaneous nephrostomy and their relationship to empirical and targeted antibiotic prophylaxis for catheter replacements* | | | | | | | | | | | | | |
| --- | --- | --- | --- | --- | --- | --- | --- | --- | --- | --- | --- | --- | --- |
|  | **PN placement** | | **Catheter replacements** | | **P^†^** | **post hoc^‡^** |  | **Empirical Atbprophylxs** | | **Targeted**  **Atbprophylxs** | | **P^†^** | **post hoc^‡^** |
|  | **N / Total** | **%** | **N / Total** | **%** |  |  |  | **N / Total** | **%** | **N / Total** | **%** |  |  |
| **Urine culture results** | | | | | <0.001 |  |  |  |  |  |  | <0.001 |  |
| *Achromobacter denitrificans* | 0 / 130 | 0% | 1 / 162 | 0.6% |  | 0.368 |  | 0 / 65 | 0% | 1 / 80 | 1.3% |  | 0.368 |
| *Achromobacter xylosoxidans* | 0 / 130 | 0% | 1 / 162 | 0.6% |  | 0.368 |  | 1 / 65 | 1.5% | 0 / 80 | 0% |  | 0.271 |
| *Acinetobacter baumannii* | 0 / 130 | 0% | 5 / 162 | 3.1% |  | 0.046 |  | 2 / 65 | 3.1% | 2 / 80 | 2.5% |  | 0.842 |
| *Aeromonas hydrophila* | 0 / 130 | 0% | 1 / 162 | 0.6% |  | 0.368 |  | 0 / 65 | 0% | 0 / 80 | 0% |  | NA |
| *Candida albicans* | 1 / 130 | 0.8% | 0 / 162 | 0% |  | 0.271 |  | 0 / 65 | 0% | 0 / 80 | 0% |  | NA |
| *Candida krusei* | 0 / 130 | 0% | 1 / 162 | 0.6% |  | 0.368 |  | 1 / 65 | 1.5% | 0 / 80 | 0% |  | 0.271 |
| *Citrobacter freundii* | 0 / 130 | 0% | 6 / 162 | 3.7% |  | 0.028 |  | 3 / 65 | 4.6% | 3 / 80 | 3.8% |  | 0.764 |
| Contaminated sample | 4 / 130 | 3.1% | 11 / 162 | 6.8% |  | 0.162 |  | 8 / 65 | 12.3% | 1 / 80 | 1.3% |  | 0.007 |
| *Enterobacter cloacae* | 1 / 130 | 0.8% | 4 / 162 | 2.5% |  | 0.271 |  | 0 / 65 | 0% | 2 / 80 | 2.5% |  | 0.194 |
| *Enterococcus faecalis* | 5 / 130 | 3.8% | 7 / 162 | 4.3% |  | 0.842 |  | 5 / 65 | 7.7% | 2 / 80 | 2.5% |  | 0.134 |
| *Enterococcus faecium* | 1 / 130 | 0.8% | 2 / 162 | 1.2% |  | 0.689 |  | 1 / 65 | 1.5% | 0 / 80 | 0% |  | 0.271 |
| *Escherichia coli* | 12 / 130 | 9.2% | 38 / 162 | 23.5% |  | 0.001 |  | 11 / 65 | 16.9% | 24 / 80 | 30% |  | 0.072 |
| *Klebsiella oxytoca* | 1 / 130 | 0.8% | 3 / 162 | 1.9% |  | 0.424 |  | 0 / 65 | 0% | 3 / 80 | 3.8% |  | 0.110 |
| *Klebsiella pneumoniae* | 8 / 130 | 6.2% | 16 / 162 | 9.9% |  | 0.230 |  | 5 / 65 | 7.7% | 11 / 80 | 13.8% |  | 0.230 |
| *Morganella morganii* | 2 / 130 | 1.5% | 17 / 162 | 10.5% |  | 0.002 |  | 5 / 65 | 7.7% | 10 / 80 | 12.5% |  | 0.368 |
| Negative | 88 / 130 | 67.7% | 10 / 162 | 6.2% |  | <0.001 |  | 8 / 65 | 12.3% | 0 / 80 | 0% |  | 0.001 |
| *Proteus mirabilis* | 0 / 130 | 0% | 3 / 162 | 1.9% |  | 0.110 |  | 2 / 65 | 3.1% | 1 / 80 | 1.3% |  | 0.424 |
| *Providencia rettgeri* | 0 / 130 | 0% | 1 / 162 | 0.6% |  | 0.368 |  | 1 / 65 | 1.5% | 0 / 80 | 0% |  | 0.271 |
| *Providencia stuartii* | 0 / 130 | 0% | 2 / 162 | 1.2% |  | 0.194 |  | 1 / 65 | 1.5% | 1 / 80 | 1.3% |  | 0.920 |
| *Pseudomonas aeruginosa* | 4 / 130 | 3.1% | 24 / 162 | 14.8% |  | <0.001 |  | 9 / 65 | 13.8% | 12 / 80 | 15% |  | 0.842 |
| *Pseudomonas luteola* | 0 / 130 | 0% | 1 / 162 | 0.6% |  | 0.368 |  | 0 / 65 | 0% | 1 / 80 | 1.3% |  | 0.368 |
| *Pseudomonas putida* | 0 / 130 | 0% | 1 / 162 | 0.6% |  | 0.368 |  | 1 / 65 | 1.5% | 0 / 80 | 0% |  | 0.271 |
| *Serratia fonticola* | 0 / 130 | 0% | 1 / 162 | 0.6% |  | 0.368 |  | 0 / 65 | 0% | 1 / 80 | 1.3% |  | 0.368 |
| *Serratia marcescens* | 0 / 130 | 0% | 5 / 162 | 3.1% |  | 0.046 |  | 0 / 65 | 0% | 5 / 80 | 6.3% |  | 0.036 |
| *Staphylococcus aureus* | 0 / 130 | 0% | 1 / 162 | 0.6% |  | 0.368 |  | 1 / 65 | 1.5% | 0 / 80 | 0% |  | 0.271 |
| *Staphylococcus haemolyticus* | 1 / 130 | 0.8% | 0 / 162 | 0% |  | 0.271 |  | 0 / 65 | 0% | 0 / 80 | 0% |  | NA |
| *Stenotrophomonas maltophilia* | 1 / 130 | 0.8% | 0 / 162 | 0% |  | 0.271 |  | 0 / 65 | 0% | 0 / 80 | 0% |  | NA |
| *Streptococcus agalactiae* | 1 / 130 | 0.8% | 0 / 162 | 0% |  | 0.271 |  | 0 / 65 | 0% | 0 / 80 | 0% |  | NA |
| **Second microorganism in urine culture results** |  |  |  |  | 0.027 |  |  |  |  |  |  | 0.693 |  |
| *Acinetobacter baumannii* | 0 / 11 | 0% | 1 / 62 | 1.6% |  | 0.689 |  | 1 / 25 | 4% | 0 / 30 | 0% |  | 0.271 |
| *Acinetobacter nosocomialis* | 1 / 11 | 9.1% | 0 / 62 | 0% |  | 0.016 |  | 0 / 25 | 0% | 0 / 30 | 0% |  | NA |
| *Candida tropicalis* | 1 / 11 | 9.1% | 0 / 62 | 0% |  | 0.016 |  | 0 / 25 | 0% | 0 / 30 | 0% |  | NA |
| *Citrobacter freundii* | 0 / 11 | 0% | 1 / 62 | 1.6% |  | 0.689 |  | 0 / 25 | 0% | 1 / 30 | 3.3% |  | 0.368 |
| Contaminated sample | 0 / 11 | 0% | 1 / 62 | 1.6% |  | 0.689 |  | 0 / 25 | 0% | 1 / 30 | 3.3% |  | 0.368 |
| *Enterobacter cloacae* | 0 / 11 | 0% | 6 / 62 | 9.7% |  | 0.271 |  | 2 / 25 | 8% | 3 / 30 | 10% |  | 0.764 |
| *Enterococcus faecalis* | 3 / 11 | 27.3% | 10 / 62 | 16.1% |  | 0.368 |  | 4 / 25 | 16% | 4 / 30 | 13.3% |  | 0.764 |
| *Enterococcus faecium* | 1 / 11 | 9.1% | 0 / 62 | 0% |  | 0.016 |  | 0 / 25 | 0% | 0 / 30 | 0% |  | NA |
| *Escherichia coli* | 2 / 11 | 18.2% | 11 / 62 | 17.7% |  | >0.999 |  | 4 / 25 | 16% | 7 / 30 | 23.3% |  | 0.484 |
| *Klebsiella pneumoniae* | 1 / 11 | 9.1% | 9 / 62 | 14.5% |  | 0.617 |  | 2 / 25 | 8% | 5 / 30 | 16.7% |  | 0.317 |
| *Morganella morganii* | 0 / 11 | 0% | 3 / 62 | 4.8% |  | 0.484 |  | 1 / 25 | 4% | 2 / 30 | 6.7% |  | 0.689 |
| *Proteus mirabilis* | 1 / 11 | 9.1% | 1 / 62 | 1.6% |  | 0.162 |  | 1 / 25 | 4% | 0 / 30 | 0% |  | 0.271 |
| *Providencia rettgeri* | 0 / 11 | 0% | 1 / 62 | 1.6% |  | 0.689 |  | 0 / 25 | 0% | 0 / 30 | 0% |  | NA |
| *Pseudomonas aeruginosa* | 0 / 11 | 0% | 13 / 62 | 21% |  | 0.089 |  | 6 / 25 | 24% | 6 / 30 | 20% |  | 0.689 |
| *Serratia marcescens* | 0 / 11 | 0% | 2 / 62 | 3.2% |  | 0.549 |  | 1 / 25 | 4% | 1 / 30 | 3.3% |  | 0.920 |
| *Staphylococcus aureus* | 0 / 11 | 0% | 3 / 62 | 4.8% |  | 0.484 |  | 3 / 25 | 12% | 0 / 30 | 0% |  | 0.046 |
| *Streptococcus agalactiae* | 1 / 11 | 9.1% | 0 / 62 | 0% |  | 0.016 |  | 0 / 25 | 0% | 0 / 30 | 0% |  | NA |
| *: Exclusion of 214 procedures due to the use of antibiotics for infection before percutaneous nephrostomy; †: Fisher's exact test; ‡: p-value adjusted by the Bonferroni method.  PN: percutaneous nephrostomy; N: absolute frequency; %: relative frequency; ; Atbprophylxs: antibiotic prophylaxis; NA: not applicable. | | | | | | | | | | | | | |

| **Supplementary Table 3.** Percutaneous nephrostomy antibiotic prophylaxis and its relationship to empirical and targeted antibiotic prophylaxis for catheter replacements* | | | | | | | | | | | | | |
| --- | --- | --- | --- | --- | --- | --- | --- | --- | --- | --- | --- | --- | --- |
|  | **PN placement** | | **Catheter replacements** | | **P**^†^ | **post hoc^‡^** |  | **Empirical Atbprophylxs** | | **Targeted**  **Atbprophylxs** | | **P**^†^ | **post hoc^‡^** |
|  | **N / Total** | **%** | **N / Total** | **%** |  |  |  | **N / Total** | **%** | **N / Total** | **%** |  |  |
| **Antibiotic used** |  |  |  |  | <0.001 |  |  |  |  |  |  | <0.001 |  |
| Amikacin | 0 / 158 | 0% | 14 / 229 | 6.1% |  | 0.001 |  | 3 / 149 | 2% | 11 / 80 | 13.8% |  | <0.001 |
| Amoxicillin-clavulanate | 1 / 158 | 0.6% | 1 / 229 | 0.4% |  | 0.764 |  | 0 / 149 | 0% | 1 / 80 | 1.3% |  | 0.162 |
| Ampicillin | 0 / 158 | 0% | 3 / 229 | 1.3% |  | 0.162 |  | 1 / 149 | 0.7% | 2 / 80 | 2.5% |  | 0.230 |
| Cefazolin | 18 / 158 | 11.4% | 5 / 229 | 2.2% |  | <0.001 |  | 5 / 149 | 3.4% | 0 / 80 | 0% |  | 0.089 |
| Ceftriaxone | 116 / 158 | 73.4% | 137 / 229 | 59.8% |  | 0.005 |  | 104 / 149 | 69.8% | 33 / 80 | 41.3% |  | <0.001 |
| Ciprofloxacin | 21 / 158 | 13.3% | 51 / 229 | 22.3% |  | 0.028 |  | 32 / 149 | 21.5% | 19 / 80 | 23.8% |  | 0.689 |
| Clindamycin | 0 / 158 | 0% | 1 / 229 | 0.4% |  | 0.424 |  | 1 / 149 | 0.7% | 0 / 80 | 0% |  | 0.484 |
| Ertapenem | 0 / 158 | 0% | 1 / 229 | 0.4% |  | 0.424 |  | 0 / 149 | 0% | 1 / 80 | 1.3% |  | 0.162 |
| Gentamycin | 2 / 158 | 1.3% | 15 / 229 | 6.6% |  | 0.012 |  | 2 / 149 | 1.4% | 13 / 80 | 16.3% |  | <0.001 |
| Trimethoprim-sulfamethoxazole | 0 / 158 | 0% | 1 / 229 | 0.4% |  | 0.424 |  | 1 / 149 | 0.7% | 0 / 80 | 0% |  | 0.484 |
| **Second antibiotic used** |  |  |  |  | 0.606 |  |  |  |  |  |  | >0.999 |  |
| Ampicillin | 1 / 3 | 33.3% | 0 / 8 | 0% |  | 0.089 |  | NA | NA | NA | NA |  |  |
| Ceftriaxone | 1 / 3 | 33.3% | 2 / 8 | 25% |  | 0.764 |  | 1 / 2 | 50% | 1 / 6 | 16.7% |  | 0.368 |
| Ciprofloxacin | 1 / 3 | 33.3% | 5 / 8 | 62.5% |  | 0.368 |  | 1 / 2 | 50% | 4 / 6 | 66.7% |  | 0.689 |
| Gentamycin | 0 / 3 | 0% | 1 / 8 | 12.5% |  | 0.549 |  | 0 / 2 | 0% | 1 / 6 | 16.7% |  | 0.549 |
| *: Exclusion of 214 procedures due to the use of antibiotics for infection before percutaneous nephrostomy; †: Fisher's exact test; ‡: p-value adjusted by the Bonferroni method.  PN: percutaneous nephrostomy; N: absolute frequency; %: relative frequency; Atbprophylxs: antibiotic prophylaxis; NA: not applicable | | | | | | | | | | | | | |

| **Supplementary Table 4.** Multiple logistic regression analyses used to adjust for potential confounders in the relationship between antibiotic prophylaxis and procedure-related UTI following the PN first placement (N=224)* | | | |
| --- | --- | --- | --- |
|  | **OR** | **95% CI** | **P** |
| Antibiotic prophylaxis | 0.000 | 0.000 | 0.993 |
| Age (in years-old) | 0.959 | 0.719-1.280 | 0.778 |
| Sex (female over male) | 0.000 | 0.000 | 0.996 |
| Neoplasms (others over cervical cancer) | 0.000 | 0.000 | 0.996 |
| UTI Treatment more than 2 weeks before PN | 224.692 | NA | 0.449 |
| Number of metastases (≥ 3 over ≤ 2) | 2.679 | 0.047-152.124 | 0.633 |
| Serum creatinine (g/dl) | 0.295 | 0.017-5.149 | 0.402 |
| Hospital (main over branch hospital) | NA | 0.000 | 0.995 |
| *: Exclusion of 214 procedures due to the use of antibiotics for infection before percutaneous nephrostomy. UTI: urinary tract infection; PN: percutaneous nephrostomy; OR: odds ratio; CI: confidence interval; ml: milliliter; KPS: Karnofsky Performance Status; g: grams; dl: deciliter. | | | |

| **Supplementary Table 5.** Multiple logistic regression analyses used to adjust for potential confounders in the relationship between antibiotic prophylaxis and procedure-related UTI following the PN catheter replacement (N=296)* | | | |
| --- | --- | --- | --- |
|  | **OR** | **95% CI** | **P** |
| Antibiotic prophylaxis | 2.412 | 0.249-23.356 | 0.447 |
| Age (in years-old) | 0.988 | 0.927-1.052 | 0.702 |
| Sex (female over male) | 1.207 | 0.088-16.541 | 0.888 |
| Neoplasms (others over cervical cancer) | 1.063 | 0.091-12.434 | 0.961 |
| Catheter-related UTI | 0.870 | 0.085-8.882 | 0.906 |
| Number of metastases (≥ 3 over ≤ 2) | 2.890 | 0.382-21.854 | 0.304 |
| Performance status – KPS (≥ 60% over up to 50%) | 0.971 | 0.119-7.895 | 0.978 |
| Uses or has used a double-J catheter | 1.670 | 0.283-9.863 | 0.571 |
| Treatment (palliative care over oncological) | 1.790 | 0.250-12.812 | 0.562 |
| Serum creatinine (g/dl) | 1.087 | 0.570-2.071 | 0.800 |
| Hospital (main over branch hospital) | 2.926 | 0.444-19.269 | 0.264 |
| *: Exclusion of 214 procedures due to the use of antibiotics for infection before percutaneous nephrostomy. UTI: urinary tract infection; PN: percutaneous nephrostomy; OR: odds ratio; CI: confidence interval; ml: milliliter; KPS: Karnofsky Performance Status; g: grams; dl: deciliter | | | |

| **Supplementary Table 6.** Sensitivity analysis performed on antibiotic prophylaxis, percutaneous nephrostomy and urinary tract infection, excluding cases without pre-procedure urine cultures* | | | | | |
| --- | --- | --- | --- | --- | --- |
|  | **Urinary tract infection** | | | | |
|  | **No** | | **Yes** | | **P^†^** |
|  | **N / Total** | **%** | **N / Total** | **%** |  |
| Procedure-related UTI |  |  |  |  |  |
| First placement | 127 / 130 | 97.7 | 3 / 130 | 2.3 | 0.520 |
| Catheter replacement | 155 / 162 | 95.7 | 7 / 162 | 4.3 |  |
|  |  |  |  |  |  |
| First placement |  |  |  |  |  |
| No antibiotic prophylaxis | 30 / 33 | 90.9 | 3 / 33 | 9.1 | 0.015 |
| With antibiotic prophylaxis | 97 / 97 | 100.0 | 0 / 97 | 0.0 |  |
|  |  |  |  |  |  |
| Catheter replacement |  |  |  |  |  |
| No antibiotic prophylaxis | 16 / 16 | 0.0 | 0 / 16 | 0.0 | >0.999 |
| With antibiotic prophylaxis | 139 / 146 | 95.2 | 7 / 146 | 4.8 |  |
|  |  |  |  |  |  |
| Empirical | 62 / 66 | 93.9 | 4 / 66 | 6.1 | 0.701 |
| Targeted | 77 / 80 | 96.3 | 3 / 80 | 3.8 |  |
| *: Exclusion of 442 procedures due to the use of antibiotics for infection before percutaneous nephrostomy and without pre-procedure urine cultures; †: Chi-squared test. N: absolute frequency; %: relative frequency; UTI: urinary tract infection; | | | | | |

**Part II – Systematic review and meta-analysis**

**Methodology**

**Search strategy**

The search strategy was structured according to the guiding question, represented by the anagram PICOS, and constructed using the Boolean operators 'OR' and 'AND' in the following databases:

- PubMed / MEDLINE: <https://pubmed.ncbi.nlm.nih.gov/>
- Cochrane library: <https://www.cochranelibrary.com/>
- EMBASE: <https://www.embase.com/landing?status=grey>
- Scopus: <https://www.scopus.com/home.uri>
- Web of science: <https://clarivate.com/academia-government/scientific-and-academic-research/research-discovery-and-referencing/web-of-science/>
- Open access theses and dissertations: <https://oatd.org/>

For PubMed, the combined terms were as follows:

(“Nephrostomy, Percutaneous” OR “Nephrostomies, Percutaneous” OR “Percutaneous Nephrostomies” OR “Percutaneous Nephrostomy” OR “Urinary Diversion” OR “Diversion, Urinary” OR “Diversions, Urinary” OR “Urinary Diversions” OR “Ileal Conduit” OR “Conduit, Ileal” OR “Conduits, Ileal” OR “Ileal Conduits” OR “Urinary Catheterization” OR “Catheterizations, Urinary” OR “Urinary Catheterizations” OR “Catheterization, Urinary” OR “Catheterization, Ureteral” OR “Catheterizations, Ureteral” OR “Ureteral Catheterizations” OR “Ureteral Catheterization” OR “Catheterization, Urethral” OR “Catheterizations, Urethral” OR “Urethral Catheterizations” OR “Urethral Catheterization” OR “Foley Catheterization” OR “Catheterization, Foley”) AND (“Urinary Tract Infections” OR “Infection, Urinary Tract” OR “Infections, Urinary Tract” OR “Tract Infection, Urinary” OR “Tract Infections, Urinary” OR “Urinary Tract Infection” OR Bacteriuria OR Bacteriurias OR Pyuria OR Pyurias OR Sepsis OR “Bloodstream Infection” OR “Bloodstream Infections” OR “Infection, Bloodstream” OR Pyemia OR Pyemias OR Pyohemia OR Pyohemias OR Pyaemia OR Pyaemias OR Septicemia OR Septicemias OR “Poisoning, Blood” OR “Blood Poisoning” OR “Blood Poisonings” OR “Poisonings, Blood” OR “Severe Sepsis” OR “Sepsis, Severe”) AND (“Antibiotic Prophylaxis” OR “Prophylaxis, Antibiotic” OR “Premedication, Antibiotic” OR “Antibiotic Premedication” OR “Antibiotic Premedications” OR “Premedications, Antibiotic” OR “Postoperative complications” OR “Complication, Postoperative” OR “Complications, Postoperative” OR “Postoperative Complication”) AND (“Ureteral Obstruction” OR “Obstruction, Ureteral” OR “Obstructions, Ureteral” OR “Ureteral Obstructions” OR Hydronephrosis OR Hydronephroses OR “Obstructive nephropathy”) AND (Neoplasms OR Neoplasia OR Neoplasias OR Neoplasm OR Tumors OR Tumor OR Cancer OR Cancers OR Malignancy OR Malignancies OR “Malignant Neoplasms” OR “Malignant Neoplasm” OR “Neoplasm, Malignant” OR “Neoplasms, Malignant”).

For EMBASE, the combined terms were as follows:

({Nephrostomy, Percutaneous} OR {Nephrostomies, Percutaneous} OR {Percutaneous Nephrostomies} OR {Percutaneous Nephrostomy} OR {Urinary Diversion} OR {Diversion, Urinary} OR {Diversions, Urinary} OR {Urinary Diversions} OR {Ileal Conduit} OR {Conduit, Ileal} OR {Conduits, Ileal} OR {Ileal Conduits} OR {Urinary Catheterization} OR {Catheterizations, Urinary} OR {Urinary Catheterizations} OR {Catheterization, Urinary} OR {Catheterization, Ureteral} OR {Catheterizations, Ureteral} OR {Ureteral Catheterizations} OR {Ureteral Catheterization} OR {Catheterization, Urethral} OR {Catheterizations, Urethral} OR {Urethral Catheterizations} OR {Urethral Catheterization} OR {Foley Catheterization} OR {Catheterization, Foley}) AND ({Urinary Tract Infections} OR {Infection, Urinary Tract} OR {Infections, Urinary Tract} OR {Tract Infection, Urinary} OR {Tract Infections, Urinary} OR {Urinary Tract Infection} OR Bacteriuria OR Bacteriurias OR Pyuria OR Pyurias OR Sepsis OR {Bloodstream Infection} OR {Bloodstream Infections} OR {Infection, Bloodstream} OR Pyemia OR Pyemias OR Pyohemia OR Pyohemias OR Pyaemia OR Pyaemias OR Septicemia OR Septicemias OR {Poisoning, Blood} OR {Blood Poisoning} OR {Blood Poisonings} OR {Poisonings, Blood} OR {Severe Sepsis} OR {Sepsis, Severe}) AND ({Antibiotic Prophylaxis} OR {Prophylaxis, Antibiotic} OR {Premedication, Antibiotic} OR {Antibiotic Premedication} OR {Antibiotic Premedications} OR {Premedications, Antibiotic} OR {Postoperative complications} OR {Complication, Postoperative} OR {Complications, Postoperative} OR {Postoperative Complication}) AND ({Ureteral Obstruction} OR {Obstruction, Ureteral} OR {Obstructions, Ureteral} OR {Ureteral Obstructions} OR Hydronephrosis OR Hydronephroses OR {Obstructive nephropathy}) AND (Neoplasms OR Neoplasia OR Neoplasias OR Neoplasm OR Tumors OR Tumor OR Cancer OR Cancers OR Malignancy OR Malignancies OR {Malignant Neoplasms} OR {Malignant Neoplasm} OR {Neoplasm, Malignant} OR {Neoplasms, Malignant})

For other databases the combination of terms was similar to PubMed.

**Results**

**Reports excluded by eligibility criteria**

**Via databases**

Participants already with urinary infection (pyelonephritis):

1. Vahlensieck, W., Friess, D., Fabry, W., Waidelich, R., & Bschleipfer, T. (2015). Long-term results after acute therapy of obstructive pyelonephritis. Urologia internationalis, 94(4), 436-441. <https://doi.org/10.1159/000368051>
2. Mert, D., Iskender, G., Kolgelier, S., & Ertek, M. (2023). Evaluation of risk factors, causative pathogens, and treatment in recurrent percutaneous nephrostomy catheter-related urinary tract infections in cancer patients. Medicine, 102(14), e33002. https://doi.org/10.1097/MD.0000000000033002

Population article duplicated with current cohort study:

1. Rodrigo Zanon, J., Cardoso, M. S., Mimica, M. J., Faria, E. F., Baiocchi, G., & Guerreiro Fregnani, J. H. T. (2020). Retrospective analysis of the role of antibiotic prophylaxis in the placement and replacement of percutaneous nephrostomy catheters in patients with malignant ureteral obstruction. Journal of Palliative Medicine, 23(5), 686-691. <https://doi.org/10.1089/jpm.2019.0289>

No information on antibiotic prophylaxis:

1. Ali, S. M., Mehmood, K., Faiq, S. M., Ali, B., Naqvi, S. A., & Rizvi, A. U. (2013). Frequency of complications in image guided percutaneous nephrostomy. JPMA. The Journal of the Pakistan Medical Association, 63(7), 816-820. PMID: 23901699.
2. Sanchez-Periut, E., Muro-Toledo, G., Losada-Guerra, J., & Reyes-Almeida, L. (2016). La nefrostomía percutánea en el carcinoma cérvico-uterino avanzado con uropatía obstructiva. Revista Mexicana de Urología, 76(4), 207-212. <https://doi.org/10.1016/j.uromx.2016.04.002>
3. Souza, A. C. P. D., Souza, A. N., Kirsztajn, R., & Kirsztajn, G. M. (2016). Cervical cancer: Renal complications and survival after percutaneous nephrostomy. Revista da Associação Médica Brasileira, 62(3), 255-261. <https://doi.org/10.1590/1806-9282.62.03.255>
4. Perri, T., Meller, E., Ben-Baruch, G., Inbar, Y., Apter, S., Heyman, L., ... & Korach, J. (2022). Palliative urinary diversion in patients with malignant ureteric obstruction due to gynaecological cancer. BMJ Supportive & Palliative Care, 12(e6), e855-e861. <https://doi.org/10.1136/bmjspcare-2019-001771>
5. Texeira, L., Pai, B. S., & Dsouza, N. (2019). Role of percutaneous nephrostomy in improving quality of life in advanced carcinoma cervix presenting with obstructive uropathy. Journal of South Asian Federation of Obstetrics and Gynaecology, 11(2), 107-109. <https://doi.org/10.5005/jp-journals-10006-1657>
6. Baio, R., Molisso, G., Perpetuini, D., Battista, E., Di Mauro, U., Intilla, O., ... & Sanseverino, R. (2025). Eternal dilemma between percutaneous nephrostomy and double J stenting in the management of patients with ureteral obstruction: A single center study. *Biomedical Reports*, *23*(1), 108. https://doi.org/ 10.3892/br.2025.1986

**Via other methods**

Article with non-oncology population:

1. Lee, W. J., Patel, U., Patel, S., & Pillari, G. P. (1994). Emergency percutaneous nephrostomy: results and complications. Journal of vascular and interventional radiology, 5(1), 135-139. <https://doi.org/10.1016/S1051-0443(94)71470-6>

Article does not evaluate urinary tract infection in results:

1. Jalbani, M. H., Deenari, R. A., Dholia, K. R., Oad, A. K., & Arbani, I. A. (2010). Role of percutaneous nephrostomy (PCN) in malignant ureteral obstruction. JPMA. The Journal of the Pakistan Medical Association, 60(4), 280-283. PMID: 20419970.

Dissertation evaluated only urine cultures:

1. Torres Viana, L. (2017). Morbimortalidad asociada a derivación percutánea de la vía urinaria en pacientes con obstrucción ureteral maligna secundaria a cáncer de cuello uterino en el Instituto Nacional de Cancerología. Departamento de Medicina Interna. Available from https://repositorio.unal.edu.co/handle/unal/59970

Dissertation conducted literature review:

1. Moström, E., & Nylander, E. (2020). Komplikationer som drabbar patienter med perkutan nefrostomi: en litteraturöversikt. Available from https://www.diva-portal.org/smash/record.jsf?pid=diva2%3A1381396&dswid=1415

No information on antibiotic prophylaxis:

1. Dudley, B. S., Gershenson, D. M., Kavanagh, J. J., Copeland, L. J., Carrasco, C. H., & Rutledge, F. N. (1986). Percutaneous nephrostomy catheter use in gynecologic malignancy: MD Anderson Hospital experience. Gynecologic oncology, 24(3), 273-278. <https://doi.org/10.1016/0090-8258(86)90303-3>
2. Van Aardt, M. C., Van Aardt, J., & Mouton, A. (2017). Impact of percutaneous nephrostomy in South African women with advanced cervical cancer and obstructive uropathy. Southern African Journal of Gynaecological Oncology, 9(1), 19-23. Available from <https://hdl.handle.net/10520/EJC-9586d0da0>

**Single-arm meta-analysis results**

**UTI rate after percutaneous nephrostomy placement with antibiotic prophylaxis**

As shown in Supplementary Figure 1, the pooled UTI rate was 9.55% (95% CI: 4.97-17.58) with I^2^=98.8%.

Leave-one-out sensitivity analysis did not explain the heterogeneity. The UTI rate in the combined population was 7.54% (95% CI: 2.81-18.70) with I^2^=87.2%, while in the oncology population it was 11.74% (95% CI: 3.70-31.54) with I^2^=92.1%.

The UTI rate in studies that did not define UTI was 9.14% (95% CI: 3.48-21.92) with I^2^=93.2%, while in studies that defined UTI it was 10.50% (95% CI: 3.13-29.92) with I^2^=71.8%.

The meta-regression model shows that follow-up time was not a source of heterogeneity among these studies (P=0.433).

The funnel plot was asymmetric, suggesting publication bias, but Eggers' test did not indicate the presence of funnel plot asymmetry (P=0.052).

**UTI rate after percutaneous nephrostomy placement without antibiotic prophylaxis**

As shown in Supplementary Figure 2, the pooled UTI rate was 7.93% (95% CI: 1.87-28.09) with I^2^=90.0%.

Leave-one-out sensitivity analysis did not explain the heterogeneity. Subgroup analyses were not performed because there was only one study that included only the oncological population (current cohort study) and only one study that did not define UTI (Cronan and colleagues(9)).

The meta-regression model shows that follow-up time was not a source of heterogeneity among these studies (P=0.465).

The funnel plot was asymmetric, suggesting publication bias, but Eggers' test did not indicate the presence of funnel plot asymmetry (P=0.583), although the number of studies was small and may lack the statistical power to detect bias.

**UTI rate after catheter replacement with antibiotic prophylaxis**

As shown in Supplementary Figure 3, the pooled UTI rate was 10.23% (95% CI: 1.75-42.11) with I^2^=96.9%.

Leave-one-out sensitivity analysis did not explain the heterogeneity. The UTI rate in the combined population was 3.47% (95% CI: 0-100) with I^2^=61.4% in two articles (Cronan and colleagues(10), and Brick and colleagues(11)), while in the oncology population it was 15.93% (95% CI: 1.20-74.69) with I^2^=97.3%.

The polled UTI rate in studies that did not define UTI was 10.62% (95% CI: 0-100) with I^2^=92.3% in two articles (Cronan and colleagues(10), and Alma and colleagues(12)) while in studies that defined UTI it was 8.45% (95% CI: 1.50-35.93) with I^2^=91.3%.

The meta-regression model shows that follow-up time was not a source of heterogeneity among these studies (P=0.329).

The funnel plot was asymmetric, suggesting publication bias, but Eggers' test did not indicate the presence of funnel plot asymmetry (P=0.126), although the number of studies was small and may lack the statistical power to detect bias.

**UTI rate after catheter replacement without antibiotic prophylaxis**

As shown in Supplementary Figure 4, the pooled UTI rate was 6.29% (95% CI: 3.30-11.68) with I^2^=0%.

Sensitivity, subgroup, and meta-regression analysis was not performed because of low heterogeneity among these three studies.

The funnel plot was asymmetric, suggesting publication bias, but Egger's test did not indicate the presence of funnel plot asymmetry (P=0.395), although the number of studies was small and may lack the statistical power to detect bias.

**Risk of bias**

Supplementary Figure 5 summarizes the ROBINS-I risk of bias assessment of the 19 studies included in the systematic review.

**Level of certainty of results**

Supplementary Table 7 summarizes the GRADE assessment.

| **Supplementary Table 7.** The level of certainty of the result determined by the GRADE assessment | | | | | | | | | | | | |
| --- | --- | --- | --- | --- | --- | --- | --- | --- | --- | --- | --- | --- |
| **Certainty assessment** | | | | | | | **№ of patients** | | **Effect** | | **Certainty** | **Importance** |
| **№ of studies** | **Study design** | **Risk of bias** | **Inconsistency** | **Indirectness** | **Imprecision** | **Other considerations** | **Antibiotic prophylaxis** | **No antibiotic prophylaxis** | **Relative (95% CI)** | **Absolute (95% CI)** |  |  |
| **Urinary tract infection related to percutaneous nephrostomy procedure (assessed with: OR)** | | | | | | | | | | | | |
| 6 | non-randomized studies | Serious* | Serious* | Serious^†^ | not serious | publication bias strongly suspected all plausible residual confounding would reduce the demonstrated effect^‡^ | 37/741 (5%) | 37/530 (7%) | **OR 0.883** (0.400 to 1.951) | **8 fewer per 1,000** (from 41 fewer to 58 more) | ⨁◯◯◯ Very low*^,†,‡^ | IMPORTANT |
| **Urinary tract infection related to first percutaneous nephrostomy catheter placement (assessed with: OR)** | | | | | | | | | | | | |
| 4 | non-randomized studies | very serious* | very serious^§^ | Serious^†^ | not serious | publication bias strongly suspected all plausible residual confounding would reduce the demonstrated effect^‡^ | 12/232 (5.2%) | 17/146 (11.6%) | **OR 0.460** (0.068 to 3.129) | **59 fewer per 1,000** (from 108 fewer to 176 more) | ⨁◯◯◯ Very low*^,†,‡,§^ |  |
|  |  |  |  |  |  |  |  |  |  |  |  |  |
|  |  |  |  |  |  |  |  |  |  |  |  |  |
| **Urinary tract infection related to percutaneous nephrostomy catheter replacement (assessed with: OR)** | | | | | | | | | | | | |
| 3 | non-randomized studies | Serious^‖^ | not serious | Serious^†^ | not serious | publication bias strongly suspected all plausible residual confounding would reduce the demonstrated effect^‡^ | 25/509 (4.9%) | 20/384 (5.2%) | **OR 1.161** (0.609 to 2.213) | **8 more per 1,000** (from 20 fewer to 56 more) | ⨁⨁◯◯ Low †^,‡ ,‖^ |  |
| CI: confidence interval; OR: odds ratio | | | | | | | | | | | | |
| Explanations: *: High heterogeneity was defined by the I-squared test with a result greater than 50%; †: Studies in oncology and non-oncology populations; ‡: Asymmetrical Funnel Plot; §: Heterogeneity by I-squared test was greater than 70%; ‖: Moderate risk of bias. | | | | | | | | | | | | |

**References**

1. Cooper FP, Alexander CE, Sinha S, Omar MIJCDoSR. Policies for replacing long‐term indwelling urinary catheters in adults. 2016(7).

2. Bierkens A, Hendrikx A, Ezz El Din K, De la Rosette J, Horrevorts A, Doesburg W, Debruyne FJEu. The value of antibiotic prophylaxis during extracorporeal shockwave lithotripsy in the prevention of urinary tract infections in patients with urine proven sterile prior to treatment. 1997;31(1):30-5.

3. Ílker Y, Türkerí LN, Korten V, Tarcan T, Akdaş AJU. Antimicrobial prophylaxis in management of urinary tract stones by extracorporeal shock-wave lithotripsy: is it necessary? 1995;46(2):165-7.

4. Claes H, Vandeursen R, Baert LJJoAC. Amoxycillin/clavulanate prophylaxis for extracorporeal shock wave lithotripsy—a comparative study. 1989;24(suppl_B):217-20.

5. Hsieh C-H, Yang SS-D, Chang S-JJSI. The effectiveness of prophylactic antibiotics with oral levofloxacin against post-shock wave lithotripsy infectious complications: a randomized controlled trial. 2016;17(3):346-51.

6. Robles J, De Castro F, Frades M, Sánchez P, Rosell D, Agüera L, et al. Prophylactic antibiotic therapy in extracorporeal shock-wave lithotripsy: prospective, randomized study. 1991;15(5):442-5.

7. Knipper A, Böhle A, Pensel J, Hofstetter AJI. Antibiotic prophylaxis with enoxacin during extracorporeal shockwave lithotripsy. 1989;17:S37-S8.

8. Mendez‐Probst CE, Goneau LW, MacDonald KW, Nott L, Seney S, Elwood CN, et al. The use of triclosan eluting stents effectively reduces ureteral stent symptoms: a prospective randomized trial. 2012;110(5):749-54.

9. Cronan JJ, Marcello A, Horn DL, Robinson A, Dorfman GS, Opal SJR. Antibiotics and nephrostomy tube care: preliminary observations: Part I. Bacteriuria. 1989;172(3):1041-2.

10. Cronan JJ, Horn DL, Marcello A, Robinson A, Paolella LP, Lambiase RE, et al. Antibiotics and Nephrostomy Tube Care: Preliminary Observations Part II. Bacteremia. 1989;172(3):1043-5.

11. Brick JM, Dail RB, Priode K, Wirth M, Yamada RJJoRN. A Practice Change to Eliminate Prophylactic Antibiotics for Elective Percutaneous Nephrostomy Tube Exchanges in Low-Risk Outpatients: A Retrospective Review on Risk for Infection. 2019;38(4):241-9.

12. Alma E, Ercil H, Vuruskan E, Altunkol A, Unal U, Gurlen G, et al. Long-term follow-up results and complications in cancer patients with persistent nephrostomy due to malignant ureteral obstruction. 2020;28:5581-8.

| **Abbreviations on main text, tables, and figures** | |
| --- | --- |
| PN | Percutaneous nephrostomy |
| UTI | Urinary tract infection |
| OR | Odds ratio |
| CI | Confidence interval |
| STROBE | Strengthening the Reporting of Observational Studies in Epidemiology |
| e.g. | *exempli gratia* |
| REDCap | Research Electronic Data Capture |
| IQR | Interquartile range |
| IBM-SPSS | International Business Machines – Statistical Package for the Social Sciences |
| PRISMA | Preferred Reporting Items for Systematic Reviews and Meta-analyses |
| PROSPERO | International Prospective Register of Systematic Reviews |
| PICOS | Anagram for Participants, Intervention, Comparisons, Outcomes, and Study design |
| MEDLINE | Medical Literature Analysis and Retrieval System Online |
| EMBASE | Excerpta Medica dataBASE |
| ROBINS-I | Risk Of Bias In Non-randomized Studies - of Interventions |
| GRADE | Grading of Recommendations Assessment, Development and Evaluation |
| GRADEpro GDT | Guideline Development Tool |
| Atbprophylxs | Antibiotic prophylaxis |
| N | Absolute frequency |
| % | Relative frequency |
| 𝑥 ̃ | Median |
| *et al.* | *et alia* |

**Tables**

**Supplementary Table 1.** Characteristics of procedures and participants who underwent percutaneous nephrostomy

**Supplementary Table 2.** Urine culture results prior to percutaneous nephrostomy and their relationship to empirical and targeted antibiotic prophylaxis for catheter replacements

**Supplementary Table 3.** Percutaneous nephrostomy antibiotic prophylaxis and its relationship to empirical and targeted antibiotic prophylaxis for catheter replacements

**Supplementary Table 4.** Multiple logistic regression analyses used to adjust for potential confounders in the relationship between antibiotic prophylaxis and procedure-related UTI following the PN first placement (N=224)

**Supplementary Table 5.** Multiple logistic regression analyses used to adjust for potential confounders in the relationship between antibiotic prophylaxis and procedure-related UTI following the PN catheter replacement (N=296)

**Supplementary Table 6.** Sensitivity analysis performed on antibiotic prophylaxis, percutaneous nephrostomy and urinary tract infection, excluding cases without pre-procedure urine cultures

**Supplementary Table 7.** The level of certainty of the result determined by the GRADE assessment

**Figures**

**Supplementary Figure 1.** Single-arm meta-analysis. **A)** Proportional meta-analysis of UTI rate after percutaneous nephrostomy placement with antibiotic prophylaxis; **B)** Leave-one-out sensitivity test; **C)** Subgroup analysis of population; **D)** Subgroup analysis of UTI definition; **E)** Meta-regression model for follow-up time in days; and **F)** Funnel plot of studies. UTI: urinary tract infection; CI: confidence interval.

**Supplementary Figure 2.** Single-arm meta-analysis. **A)** Proportional meta-analysis of UTI rate after percutaneous nephrostomy placement without antibiotic prophylaxis; **B)** Leave-one-out sensitivity test; **C)** Meta-regression model for follow-up time in days; and **D)** Funnel plot of studies. UTI: urinary tract infection; CI: confidence interval.

**Supplementary Figure 3.** Single-arm meta-analysis. **A)** Proportional meta-analysis of UTI rate after catheter replacement with antibiotic prophylaxis; **B)** Leave-one-out sensitivity test; **C)** Subgroup analysis of population; **D)** Subgroup analysis of UTI definition; **E)** Meta-regression model for follow-up time in days; and **F)** Funnel plot of studies. UTI: urinary tract infection; CI: confidence interval.

**Supplementary Figure 4.** Single-arm meta-analysis. **A)** Proportional meta-analysis of UTI rate after catheter replacement without antibiotic prophylaxis; **B)** Funnel plot of studies. UTI: urinary tract infection; CI: confidence interval.

**Supplementary Figure 5.** ROBINS-I risk of bias assessment. **A)** Traffic light plot. **B)** Summary plot
